# Compromised Endothelial Regeneration as a Potential Contributor to Long-Term Cardiovascular Complications in Childhood Cancer Survivors

**DOI:** 10.3390/cells15212040

**Published:** 2026-11-09

**Authors:** Kasturi Markandran, Tricia Jingwen Ng, Elena Tan, Czaryna Kaye Mendoza Clemente, Rina Miao Qin Wang, Yee Phong Lim, Karan Attal, Kristine Nicole Mendoza Clemente, Hammresh S, Sik Yin Roger Foo, Ching Kit Chen

**Affiliations:** 1 Department of Paediatrics, Yong Loo Lin School of Medicine, National University of Singapore, Singapore 119228, Singapore; kas_mark@nus.edu.sg (K.M.); e0968526@u.nus.edu (T.J.N.); czarynac@nus.edu.sg (C.K.M.C.); rinawmq@nus.edu.sg (R.M.Q.W.); lin.yipeng@hotmail.com (Y.P.L.); kristinenicole.clemente@u.nus.edu (K.N.M.C.); 2607hammresh@gmail.com (H.S.); 2 Cardiovascular Research Institute, Department of Medicine, Yong Loo Lin School of Medicine, National University of Singapore, National University Health System, Singapore 119228, Singapore; roger.foo@nus.edu.sg; 3 Royal College of Surgeons in Ireland (Medicine), School of Medicine, Royal College of Surgeons in Ireland, D02 YN77 Dublin, Ireland; elenatan20@rcsi.ie (E.T.); karanattal21@rcsi.ie (K.A.); 4 Cardiovascular Disease Translational Research Programme, Yong Loo Lin School of Medicine, National University of Singapore, Singapore 119228, Singapore; 5 Division of Cardiology, Department of Paediatrics, Khoo Teck Puat–National University Children’s Medical Institute, National University Health System, Singapore 119228, Singapore

**Keywords:** circulating endothelial cells, endothelial progenitor cells, endothelial injury, senescence, leukaemia, lymphoma

## Abstract

Anthracyclines are central to childhood cancer therapy but predispose patients to cardiovascular toxicity and long-term cardiovascular risk. Endothelial injury and impaired repair contribute to this, yet paediatric data remain limited. This study longitudinally assessed endothelial injury and repair by quantifying circulating endothelial cells (CECs) and endothelial progenitor cells (EPCs). In a single-centre prospective cohort, children (<18 years) with leukaemia (*n* = 35) or lymphoma (*n* = 13) were studied at four timepoints: pre-treatment (Pre), ~1 month post-treatment (End), 3 months (3M), and 1 year (1Y). Blood mononuclear cells were analysed by flow cytometry to quantify CECs and EPCs, and EPC fate by p16 (senescence) and Annexin V (apoptosis). Cardiac biomarkers and left ventricular function were assessed. Longitudinal trends of CEC and EPC counts were similar between leukaemia and lymphoma participants. CECs were highest at pre-treatment and declined significantly thereafter, though they remained marginally elevated during remission compared with healthy controls, indicating endothelial damage had largely subsided following treatment. EPCs were highest at pre-treatment and decreased below healthy controls during remission, suggesting impaired baseline endothelial maintenance and repair. While EPC fate did not alter significantly from healthy controls before and after treatment in leukaemia patients, in lymphoma patients the EPCs were pre-dominantly senescent right after treatment completion and apoptotic activity gradually returned to healthy control levels, suggesting reduced EPC involvement in tumour-associated angiogenesis following treatment and persistent impairment of endothelial regenerative capacity, although their relative contributions cannot be determined from the present study. In summary, this study demonstrates that endothelial injury largely subsides following treatment, but regeneration, and hence repair, remains impaired during remission.

## 1. Introduction

Anthracycline is a class of chemotherapeutic agents used to treat more than half of all childhood cancers [1]. Despite their effectiveness in treating cancers, anthracyclines are associated with inducing cytotoxicity in other cell types resulting in adverse outcomes [2]. Given that endothelial cells lining the blood vessels serve as a protective barrier for cardiac cells and are the most abundant cell type in the heart, their impairment and cell death can exacerbate acute and late-onset anthracycline-mediated cardiac injury [3,4,5,6,7,8]. It is well-established that endothelial dysfunction increases the risk of cardiovascular complications [9]. Thus, to assess endothelial health, we longitudinally enumerated circulating endothelial cells (CECs) and endothelial progenitor cells (EPCs) in the peripheral circulation of paediatric leukaemia and lymphoma patients who were treated with anthracyclines. CEC is a putative marker of endothelial injury, while EPC is regarded as marker of endothelial repair and neovascularization [10,11,12]. In addition, cardiac injury markers present in plasma such troponin T (TnT) and N-terminal pro B-type natriuretic peptide (NT-proBNP) were assayed to elucidate the potential impact of endothelial health on anthracycline-mediated cardiac injury.

Leukaemia begins in the bone marrow where hematopoietic cells proliferate aberrantly into immature forms [13]. The uncontrolled proliferation exacerbates hypoxia in the bone marrow triggering hypoxic-inducible factors, which stimulate angiogenesis and mobilize EPCs into the circulation [14,15,16]. Furthermore, systemic inflammation in leukemic patients can trigger the mobilization of EPCs from the bone marrow to the site of tissue injury [15,17]. Other potential sources of circulating EPCs include vascular resident endothelial cells and tissue vascular niches [18]. In the setting of cancer, EPCs are indicative of either vascular repair or severity of cancer and need to be interpreted with caution [19]. Separately, high CEC counts have been reported in adult leukaemia patients when compared to treated patients and healthy controls [20,21], potentially due to systemic inflammation [22,23] or the disruption of existing vasculature (generating CECs) facilitating angiogenesis in the bone marrow [16,24]. However, to date, no studies have longitudinally monitored CEC and EPC counts in childhood leukaemia patients up to one-year post-treatment, a period during which early-onset cardiotoxicity often develops [25].

Lymphoma develops in the lymphatic system [26]. The progression of lymphoma is enabled by vascularization, also known as “angiogenic switch”, and hence recruit EPCs to the cancer microenvironment [27,28]. The inflammatory-laden setting and disruption of blood vessels to facilitate angiogenesis can cause CECs to be elevated [29,30]. However, there are no reports of CEC and EPC counts on acute childhood lymphoma.

In addition to enumerating EPCs logitudinally, the proportion of EPCs committed to the senescent and apoptotic fates were characterized in both leukaemia and lymphoma patients. Emerging evidence indicates that anthracyclines trigger both apoptotic and senescent pathways in EPCs, both of which impair EPC function [31]. EPC fates will shed light on their capability on maintaining vascular health. While the effect of EPC cell fate on acute- and late-onset cardiotoxicity has not been explored before, the restoration of EPC function ameliorated doxorubicin-induced cardiac dysfunction in a rat model [6]. Thus, we characterized EPC cell fates, specifically apoptotic and senescent EPCs, in anthracycline-treated paediatric patients.

This study is the first to report longitudinal trends in CECs, EPCs, and cardiomyocyte injury biomarkers in childhood cancer patients from the time of diagnosis to one-year post-anthracycline treatment. Additionally, the cell fate of EPCs was characterized to assess its usefulness in facilitating endothelial health. Having a comprehensive understanding of the course of endothelial health and injury will shed light on the initial asymptomatic insult leading to late-onset cardiovascular complications [8,32]. These markers have the potential to guide diagnosis and prognosis [33] and are necessary with the increasing numbers of childhood cancer survivors [34].

## 2. Materials and Methods

### 2.1. Study Population

This was a single-centre, prospective observational study that was approved by the institutional research ethics board, the Domain Specific Review Board of National Healthcare Group, Singapore. Eligible participants including children and adolescents (<18 years old) diagnosed with cancer who were due to receive anthracyclines as part of combination chemotherapy were recruited between 2019 and 2024 at the National University Hospital (NUH). Only children with normal cardiac function (LVEF ≥ 55%) prior to initiation of anthracycline therapy were included in this study; patients with congenital heart disease (except patent foramen ovale/small atrial septal defect) were excluded. Clinical data were obtained from the patients’ electronic medical record. Peripheral blood and echocardiograms were obtained at four timepoints for this study: (1) before anthracycline treatment (“Pre”), (2) one month after completion of treatment (“End”), (3) three months after end of treatment (“3M”), and (4) one year after end of treatment (“1Y”). Healthy control subjects were made up of paediatric patients referred for heart murmur assessment who had normal results on echocardiography, physical examination, and electrocardiography, and from a group of healthy volunteers.

### 2.2. Blood Processing

Approximately 10 mL of blood was collected in EDTA tubes at each timepoint and centrifuged at 4000 rpm for 10 min at 4 °C within 2 h of collection. Plasma was transferred to a clean tube and gently resuspended to get a homogenous mixture before transferring to cryotubes for storage. Subsequently, buffy coats were transferred into cryotubes. Both plasma and buffy coats were stored at −80 °C until analysis.

### 2.3. Circulating Endothelial Cell and Endothelial Progenitor Cell Enumeration via Flow Cytometry

To recover mononuclear cells, frozen buffy coats were thawed and diluted threefold with phosphate-buffered saline (PBS). The diluted buffy coat was layered over a half volume of Ficoll-Paque™ PLUS (Cytiva Sweden AB, Uppsala, Sweden) density gradient media and centrifuged at 1000× *g* for 20 min with an acceleration setting at 1 and no deceleration, at room temperature. The layer of mononuclear cells was transferred to a new tube, mixed with an equal volume of PBS, and subsequently centrifuged at 540× *g* for 7 min at maximum acceleration and deceleration. The resulting cell pellet was washed with 5 mL of PBS and centrifuged at the same setting. The cell pellet was resuspended in 500 µL of 1% bovine serum albumin (BSA) in PBS for cell counting. Aliquots of cells were distributed into individual tubes for antibody staining, and remaining cells were cryopreserved for future use. Each tube of cells was stained with 4 antibodies: CD45 (FITC), CD31 (PE-Cy7), CD133 (APC), and Hoechst 33342. Following 10 min incubation at room temperature, the cells were incubated in the cold room (~4 °C) on a see-saw rocker for 20 min. After incubation, 1 mL of 1% BSA in PBS was added to the cells to dilute excess antibodies, followed by centrifugation at 300× *g* for 5 min at 4 °C to remove them. The resulting cell pellet was resuspended in 200 µL of 1% BSA in PBS and filtered through a 60 µm filter to prevent cell clumps, yielding a single-cell suspension. The cell suspension was analysed using the Beckman Coulter CytoFLEX LX Flow Cytometer (Beckman Coulter, Inc., Brea, CA, USA), with 10,000 cells analysed per tube. CECs were identified based on the immunophenotypic profile CD45^−^/CD31^+^/CD133^−^/DNA^+^, while EPCs were identified by CD45^−^/CD31^+^/CD133^+^. If fewer than 10,000 total cells are acquired, the number of cells of interest will be normalised to the total number of cells acquired.

### 2.4. Endothelial Progenitor Cell-Fate Characterization via Flow Cytometry

Using the remaining cryopreserved cells, EPCs were further characterized for apoptosis using Annexin V staining, and senescence using p16 staining. Annexin V antibody was conjugated to Pacific Blue fluorophore with an emission wavelength overlapping that of Hoechst 33342. Therefore, Hoechst staining was excluded during the characterization of apoptotic EPCs, which were identified using the immunophenotypic profile CD45^−^/CD31^+^/CD133^+^/Annexin V^+^. Annexin V binds to phosphatidylserine on the cell surface in a calcium-dependent manner, necessitating the use of a calcium-containing binding buffer. The cells were first stained with CD45, CD31, and CD133 antibodies as described in Section 2.3. Following the final centrifugation, the resulting cell pellet was resuspended in 100 µL of binding buffer (BioLegend #422201) followed by the addition of Annexin V antibodies. The cells were incubated for 15 min at room temperature in the dark to facilitate binding. Finally, 200 µL of binding buffer was added to achieve a volume of 300 µL for flow cytometric analysis. p16 antibody was conjugated to CoraLite^®^555. Senescent EPCs were identified by an immunophenotypic profile of CD45^−^/CD31^+^/CD133^+^/p16^+^. To achieve this, the cells were first stained with all antibodies, except p16, as described above. Prior to staining with p16 antibody, the cells were fixed by incubating the cells in 500 µL of fixation buffer (BioLegend #420801) for 20 min at room temperature. Following this, the cells were centrifuged at 350× *g* for 5 min and subsequently resuspended in 500 µL of 1× permeabilization and wash buffer (BioLegend #421002). This permeabilization step was repeated once more. After the final centrifugation, most of the supernatant was removed, leaving 100 µL of residual buffer. Cells were resuspended in the residual buffer and 1 µL of p16 antibody was added. The cells were incubated for 20 min at room temperature in the dark. Following incubation, 1 mL of 1× permeabilization and wash buffer were added to dilute unbound antibodies, and the cells were centrifuged at 350× *g* for 5 min. The pellet was resuspended in 300 µL of 1× permeabilization and wash buffer and filtered through a 60 µm filter to remove cell clumps. The single-cell suspensions were analysed using the Beckman Coulter CytoFLEX LX Flow Cytometer, with 10,000 cells analysed per tube. If fewer than 10,000 total cells are acquired, the number of cells of interest will be normalised to the total number of cells acquired.

### 2.5. Transthoracic Echocardiography

Transthoracic echocardiography was performed using a Lisendo 880 echocardiographic system (Fujifilm Healthcare Co., Ltd., Kashiwa, Japan) using a standard clinical protocol, in accordance with published guidelines [35]. Echocardiographic parameters were analysed offline using TomTec vendor-independent analysis software (TomTec, Chicago, IL, USA). Left ventricular ejection fraction (LVEF) was determined using the biplane Simpson method and was used to assess left ventricular systolic function throughout the study period.

### 2.6. Cardiac Protein Assay

High-sensitivity cardiac troponin T (hsTnT) assay and N-terminal pro-B-type natriuretic peptide (NTproBNP) assay were performed using the Roche Diagnostics, Cobas e411 automated analyser (Roche Diagnostics GmbH, Mannheim, Germany) using 300 µL of plasma from the respective timepoints. TnT is released into the peripheral circulation upon cardiomyocyte injury, thus becoming a robust marker of cardiomyocyte damage [36]. NTproBNP is synthesized and secreted by ventricular cardiomyocytes in response to increased ventricular wall stress, making it a robust marker of ventricular dysfunction [37].

### 2.7. Statistical Analysis

Statistical analysis was performed using RStudio 2026.07.1 Build 147 or a commercially available package, GraphPad Prism 7.2 (GraphPad Software, San Diego, CA, USA). Longitudinal changes were analysed using linear mixed-effects models (LMMs), with timepoint included as a fixed effect and participant identity as a random intercept to account for repeated measurements. Initially, models assuming homogeneous and heterogeneous residual variances across timepoints were fitted using untransformed data. Residual normality was assessed using the Shapiro–Wilk test. Where significant deviations from normality were observed, the data were log-transformed, and both models were refitted. Models assuming homogeneous and heterogeneous residual variances were compared using likelihood-ratio tests and the Akaike information criterion (AIC). The final model was selected based on model fit, parsimony, and residual diagnostics, including a reassessment of normality using the Shapiro–Wilk test. The overall effect of timepoint was assessed using a marginal F-test, with healthy controls (HCs) excluded from this analysis to evaluate longitudinal changes in patients. A separate model incorporating HCs was used to evaluate differences between individual timepoints and HCs. Pairwise comparisons were performed using estimated marginal means, with Holm–Šidák for multiple comparisons.

For proportional outcomes (p16^+^ and Annexin^+^ cell proportions) containing zero values that did not adequately satisfy LMM assumptions, ordered beta mixed-effects models were fitted using the glmmTMB package. If residuals were normally distributed, a raw-scale linear mixed-effects model with a patient random intercept was used. Timepoint was included as a fixed effect and participant identity as a random intercept. The overall effect of timepoint was evaluated by comparing models with and without the timepoint fixed effect using likelihood-ratio tests, with healthy controls (HCs) excluded from this analysis. Pairwise comparisons between timepoints were performed using estimated marginal means (emmeans) on the on the logit (link) scale, with Holm–Šidák or Tukey correction for multiple comparisons, where appropriate. More details are provided in the figure legends.

All data are shown as mean ± S.E.M of the raw data. All statistical output were graphically presented on their original, untransformed scale. A confidence level of *p* < 0.05 was considered statistically significant.

## 3. Results

### 3.1. Demographics and Clinical Parameters of Patients

Thirty-five patients diagnosed with leukaemia were recruited. Among these, 78% had acute lymphoblastic leukaemia (ALL) and the remaining 22% had acute myeloid leukaemia (AML). Separately, 13 patients diagnosed with lymphoma were recruited; of these, 38% were diagnosed with Hodgkin Lymphoma, 38% Burkitt lymphoma, and the remainder with anaplastic large-cell, B-cell, or primary mediastinal B-cell lymphoma. Age-matched comparisons were performed for CEC and EPC enumeration experiments. Anthracycline cumulative dose and left ventricular ejection fraction (LVEF) are similar in both leukaemia and lymphoma participants across all timepoints of theis study (Table 1, Figure S1). All the patients were in remission by the end of treatment, based on the lack of blast cells in bone marrow for leukaemia patients (determined by bone marrow aspiration) and lack of active foci of lymphoma via PET-CT data.

Separately, neutrophil-to-lymphocyte ratio (NLR) was used to assess systemic inflammation. Although the threshold for childhood cancers is not established [38,39], typically, NLR > 3 reflects systemic inflammation. Based on this, pronounced inflammation was observed at 3 months post-treatment (NLR = 5.7) in participants with leukaemia and at pre-treatment (NLR = 7.4) in those with lymphoma (Table 2).

### 3.2. Peripheral CEC and EPC Enumeration in Leukaemia Patients

**Figure 1:**
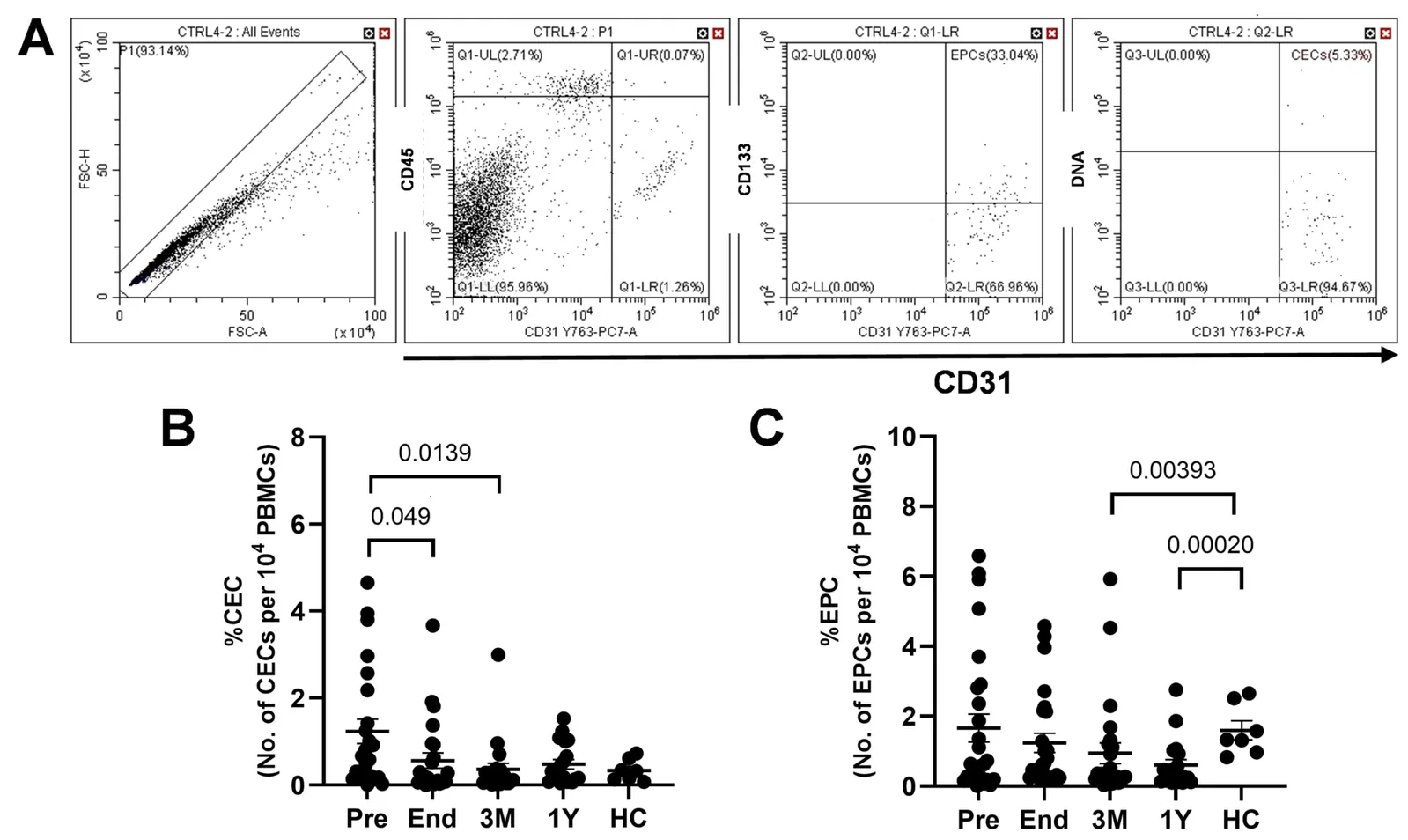
(**A**) Gating strategy employed for flow cytometry result to identify CECs and EPCs. (**B**) **Enumeration of CECs in leukemic patients at pre-treatment (*n* = 24), end treatment (*n* = 24), 3 months post-treatment (*n* = 21), 1 year post-treatment (*n* = 19), and healthy controls (*n* = 7).** %CECs in 10,000 PBMCs. (**C**) **Enumeration of EPCs in leukemic patients at pre-treatment (*n* = 27), end treatment (*n* = 25), 3 months post-treatment (*n* = 24), 1 year post-treatment (*n* = 19), and healthy controls (*n* = 7).** %EPCs in 10,000 PBMCs. Data are presented as a scatterplot of mean ± SEM. %CEC and %EPC were log-transformed because both homogenous- and heterogenous-variance raw-scale models showed departures from residual normality. Pairwise comparisons, including HCs, were also performed on log-transformed data and were Holm–Šídák adjusted. Numerical values and raw data are presented in the Supplementary Materials (Table S1 and Excel file).

CECs were in the range between 0.35% and 1.2% of 10,000 peripheral blood mononuclear cells (PBMCs). Levels were highest at the time of diagnosis (i.e., pre-treatment) where CEC counts were approximately four-fold higher than healthy controls. CECs then decreased after treatment (i.e., End, 3M, and 1Y timepoints) to levels comparable to healthy controls, although remaining marginally higher (Figure 1B). This indicates that the processes of cancer itself caused more endothelial damage than the residual effects of anthracycline treatment. The log-transformed linear mixed-effects model, with timepoint as a fixed effect and patient as a random intercept, showed statistically significant changes across the four timepoints (F(3,54) = 4.31, *p* = 0.0085).

The circulating EPC count is between 0.6% and 1.6% in 10,000 PBMCs. EPC counts at the pre-treatment timepoint were comparable to healthy controls. However, the counts gradually decreased through End, 3M, and 1Y timepoints, and were generally lower when compared to healthy controls, implying that endothelial repair is impaired after treatment (Figure 1C). Collectively, these findings suggest that persistent endothelial impairment during remission is more likely attributable to defective repair mechanisms than to active residual chemotherapy-induced injury. The log-transformed linear mixed-effects model, with timepoint as a fixed effect and patient as a random intercept, showed that the changes across the four timepoints were not statistically significant (F(3,58) = 2.49, *p* = 0.0692).

### 3.3. Endothelial Progenitor Cell-Fate Characterization in Leukaemia Patients

**Figure 2:**
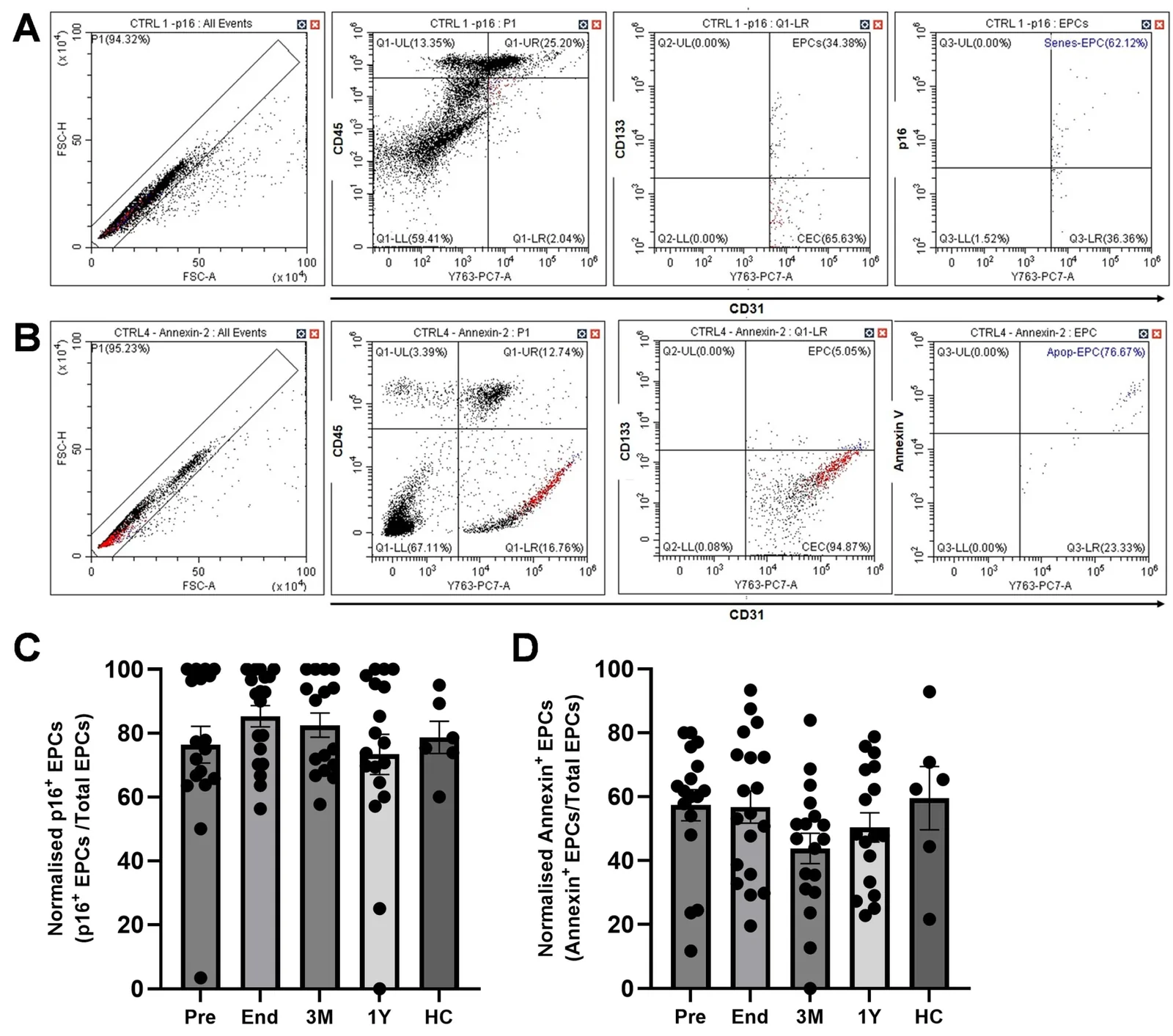
(**A**) Gating strategy employed for flow cytometry data to identify senescent EPCs. (**B**) Gating strategy employed for flow cytometry data to identify apoptotic EPCs. (**C**)** Senescent (p16^+^) EPC enumeration in leukemic patients at pre-treatment (*n* = 18), end treatment (*n* = 19), 3 months post-treatment (*n* = 16), 1 year post-treatment (*n* = 18), and healthy controls (*n* = 6).** Percentage of EPCs positive for the p16 signal relative to total EPCs. As raw-scale models violated residual normality assumptions and the data contained zero values that precluded direct log-transformation, an ordered beta mixed-effects model was used. Pairwise comparisons, including HCs, were adjusted for multiple testing using the Holm–Šídák method. (**D**) **Apoptotic EPC enumeration in leukemic patients at pre-treatment (*n* = 17), end treatment (*n* = 19), 3 months post-treatment (*n* = 18), 1 year post-treatment (*n* = 17), and healthy controls (*n* = 6).** Percentage of EPCs with a positive Annexin V signal relative to total EPCs. As raw-scale models showed residual normality, a raw-scale linear mixed-effects model with a patient random intercept was used. Pairwise comparisons, including HCs, were adjusted for multiple testing using the Tukey method. Data are presented as a scatterplot of mean ± SEM. Numerical values and raw data are presented in the Supplementary Materials (Table S1 and Excel).

While the decrease in EPCs in the peripheral blood circulation between End and 1Y timepoints (Figure 1C) itself contributes to disrupted endothelial repair, it is important to understand the viability of the available cells to comprehensively evaluate their reparative capacity. This is because the cytotoxic effects of anthracyclines trigger apoptotic and senescent pathways in cells [40,41]. Thus, we examined the fraction of EPCs that committed to the senescent or apoptotic pathway via flow cytometry (Figure 2A,B). In this study, the senescent p16^+^ EPCs were in the range between 73% and 85% of all EPCs detected (Figure 2C). p16^+^ EPCs were comparable to healthy controls at all timepoints in leukaemia participants. The ordered beta mixed-effects model with timepoint as a fixed effect and patient as a random intercept was used, and there were no statistically significant changes across the four timepoints (likelihood-ratio χ^2^(3) = 2.22, *p* = 0.529).

The apoptotic Annexin V^+^ cells were in the range between 44% and 57% of all EPCs detected. Unlike senescent EPCs, the proportion of apoptotic EPCs decreased by 30% at 3 months when compared to healthy controls (although not statistically significant) (Figure 2D). The decrease in apoptotic cells at 3 months post-treatment could be attributed to the nature of anthracycline chemotherapy as doxorubicin-induced apoptosis is reported to occur acutely [42], suggesting that anthracycline-induced apoptosis of EPCs potentially subsides by 3 months post-treatment. Additionally, the reduction in apoptotic EPCs may have resulted from increased cellular senescence, particularly at 3 months post-treatment, resulting in apoptotic EPCs to decrease below healthy baseline, as senescent cells often upregulate anti-apoptotic pathways that counteract apoptosis [43]. However, the linear mixed-effects model showed that the changes in the proportion of apoptotic EPCs across the four timepoints were not statistically significant (F(3,41) = 1.75, *p* = 0.172). It is notable that baseline EPC apoptosis in healthy controls is comparable with cancer- and anthracycline-induced apoptosis, at Pre and End timepoints, respectively. While there is no literature that supports this theory, we postulate that a high proportion of apoptotic EPCs in healthy controls could be a regulatory response that limits excessive vascular regeneration or angiogenesis, which could otherwise support tumour growth. Regardless, these data demonstrate that a significant proportion of circulating EPCs develop senescence after treatment, suggesting that endothelial impairment during remission could potentially be due to defective repair mechanisms.

### 3.4. Cardiac Protein Biomarkers in Leukaemia Patients

**Figure 3:**
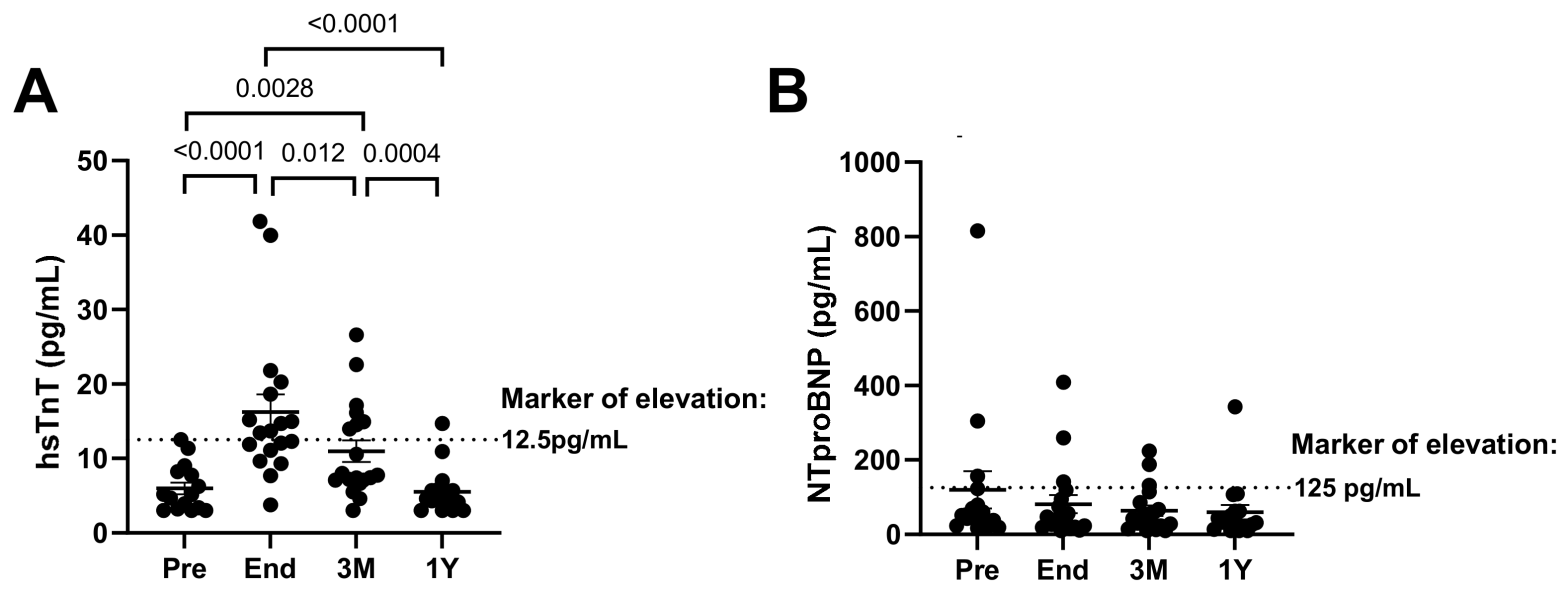
(**A**) **Assay of high-sensitivity troponin T (hsTnT) in leukemic patients at pre-treatment (*n* = 15), end treatment (*n* = 18), 3 months post-treatment (*n* = 19), and 1 year post-treatment (*n* = 17).** Data were log-transformed due to non-normal residuals in both raw-scale models. Although residual normality remained unsatisfactory after transformation (*p* = 0.037), the homogeneous-variance model showed improved residual normality and was selected based on its lower AIC. Pairwise comparisons were adjusted for multiple testing using the Holm–Šídák method. (**B**) **Assay of NTproBNP in leukemic patients at pre-treatment (*n* = 16), end treatment (*n* = 18), 3 months post-treatment (*n* = 19), and 1 year post-treatment (*n* = 17).** Data were log-transformed due to non-normal residuals in both raw-scale models. Although residual normality remained unsatisfactory after transformation, it improved in both models (*p* > 0.01). The homogeneous-variance model was selected based on its lower AIC. Pairwise comparisons were adjusted for multiple testing using the Holm–Šídák method. Data are presented as a scatterplot of mean ± SEM. Numerical values and raw data are presented in the Supplementary Materials (Excel).

In the majority of patients, hsTnT levels were within the normal limit before cancer treatment (5.98 ± 0.81 pg/mL). However, hsTnT levels were significantly elevated at the end-treatment stage (16.2 ± 2.3 pg/mL), indicating a significant increase in cardiomyocyte damage. The levels decreased from End to 3 months post-treatment (11.0 ± 1.5 pg/mL) with 37% of the participants remaining above the marker of elevation. By 1 year post-treatment, the hsTnT levels of almost all participants returned to normal levels (5.53 ± 0.74) (Figure 3A). A linear mixed-effects model, with timepoint as a fixed effect and patient as a random intercept, showed that the changes across the four timepoints were statistically significant (F(3,46) = 20.76, *p* < 0.0001).

On the other hand, NTproBNP levels of the participants were mostly in the acceptable range throughout the study period, implying that there was no significant cardiac remodelling despite cardiomyocyte injury (Figure 3B). The linear mixed-effects model, with timepoint as a fixed effect and patient as a random intercept, found no statistical significance across the four timepoints (F(3,47) = 1.29, *p* = 0.287). This finding is further supported by the preservation of LVEF within the normal range of these participants (Figure S1). Spearman test was performed to identify the correlation between hsTnT (cardiomyocyte damage) and CECs (endothelial damage) and hsTnT and EPCs, respectively. However, there were no significant correlations revealed (Figure S2). The main limitation of this approach lies in the non-organ-specific nature of CECs. While higher CEC levels suggest increased endothelial shedding, it cannot be definitively concluded that these cells originate from the coronary vasculature, thereby limiting the accuracy of linking cardiac endothelial injury to cardiomyocyte damage. Hence, using organ-specific EC injury markers like cell-free DNA will be useful to elucidate whether damage to cardiac- or coronary-specific endothelium exacerbates cardiomyocyte damage or vice versa.

### 3.5. Peripheral CEC and EPC Enumeration in Lymphoma Patients

**Figure 4:**
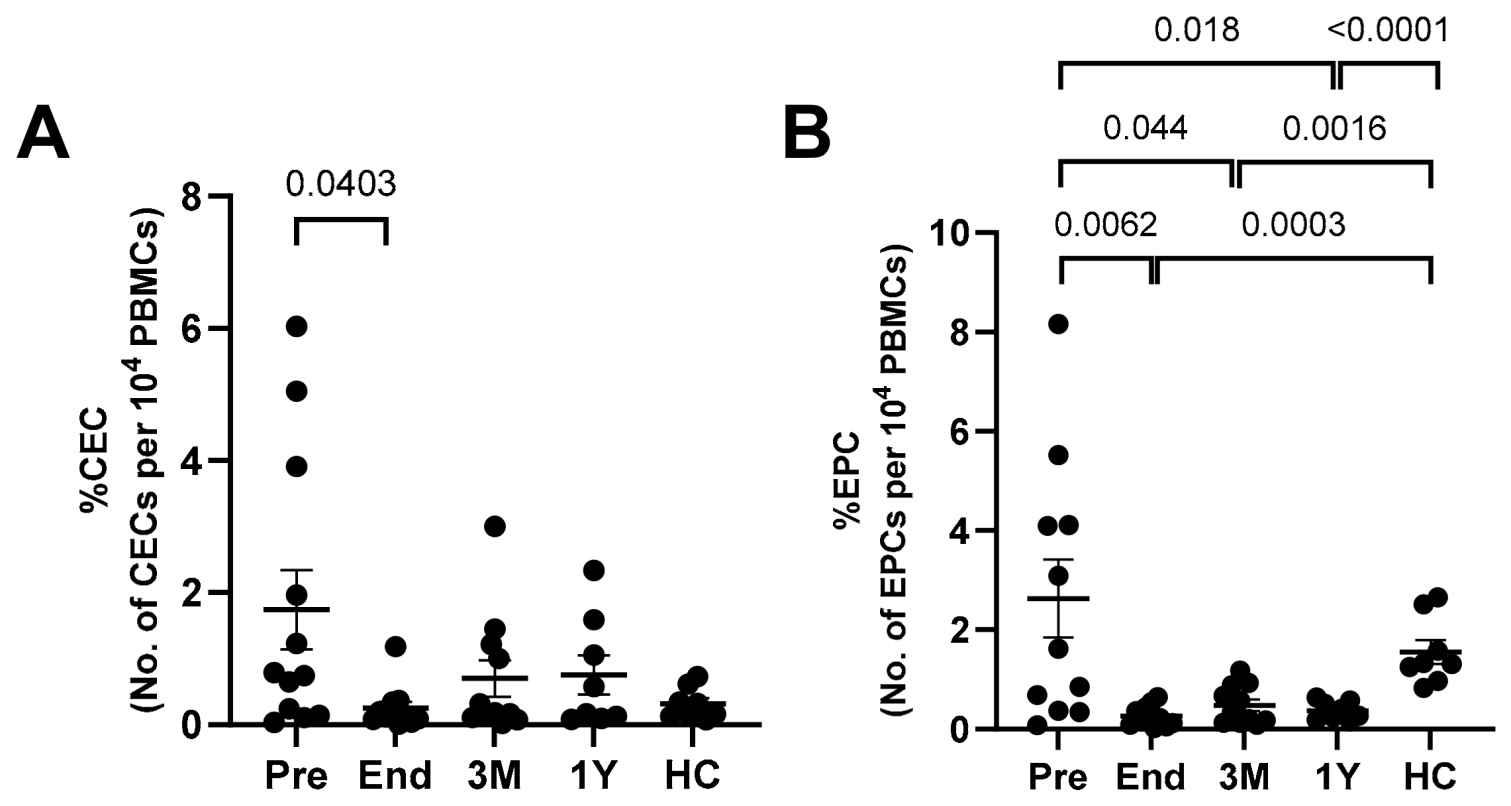
(**A**) **Enumeration of CECs in lymphoma patients at pre-treatment (*n* = 12), end treatment (*n* = 11), 3 months post-treatment (*n* = 11), 1 year post-treatment (*n* = 8), and healthy controls (*n* = 8).** %CECs in 10,000 PBMCs. %CEC was log-transformed due to non-normal residuals in both raw-scale models and subsequently analysed using a linear mixed-effects model. Pairwise comparisons, including HCs, were also performed on log-transformed data and were Holm–Šídák adjusted. (**B**) **Enumeration of EPCs in lymphoma patients at pre-treatment (*n* = 11), end treatment (*n* = 11), 3 months post-treatment (*n* = 11), 1 year post-treatment (*n* = 8), and healthy controls (*n* = 8).** %EPCs in 10,000 PBMCs. %EPC was log-transformed due to non-normal residuals in both raw-scale models and subsequently analysed using a linear mixed-effects model. Pairwise comparisons, including HCs, were also performed on log-transformed data and were Holm–Šídák adjusted. Data are presented as a scatterplot of mean ± SEM. Numerical values and raw data are presented in the Supplementary Materials (Table S2 and Excel).

CECs were in the range between 0.25% and 1.74% in 10,000 PBMCs. Just as in leukaemia participants, lymphoma patients exhibited the highest CEC levels at diagnosis, with counts approximately 5.5-fold higher than those of healthy controls (Figure 4A). This elevation was consistent with the notion that endothelial disruption is required to support angiogenesis during lymphoma progression. Additionally, depending on the cancer severity, an inflammatory-laden environment may exacerbate vascular injury, contributing to CECs [44]. Enhanced inflammation at pre-treatment was reflected by the NLR (Table 2). CEC levels decreased to levels comparable to healthy controls at the end of treatment, but subsequently increased to approximately threefold higher than healthy control levels at 3 months and 1 year post-treatment, albeit without statistical significance (Figure 4A). This suggests that persistent endothelial impairment is attributable to residual anthracycline-induced endothelial injury. The log-transformed linear mixed-effects model, with timepoint as a fixed effect and patient as a random intercept, showed statistical significance across the four timepoints (F(3,26) = 3.03, *p* = 0.0472).

Due to the nature of lymphoma, circulating EPCs may reflect not only endothelial repair but also cancer-driven angiogenesis, and thus serve as an indicator of cancer progression. The circulating EPC count ranges between 0.26% and 2.6% in 10,000 PBMCs (Figure 4B). However, the counts were significantly decreased at End, 3M, and 1Y timepoints when compared to pre-treatment and healthy controls. The significant decrease indicates that the progression of lymphoma is impeded, which corroborates with negative findings in the PET-CT imaging of the participants. However, a significant decrease compared to healthy controls indicates that basal endothelial repair is impaired. The log-transformed linear mixed-effects model, with timepoint as a fixed effect and patient as a random intercept, showed a statistically significant overall timepoint effect (F(3,25) = 4.83, *p* = 0.0087). Collectively, the CEC and EPC profiles suggest that persistent endothelial impairment during remission is driven predominantly by reduced reparative capacity.

### 3.6. Endothelial Progenitor Cell-Fate Characterization in Lymphoma Patients

**Figure 5:**
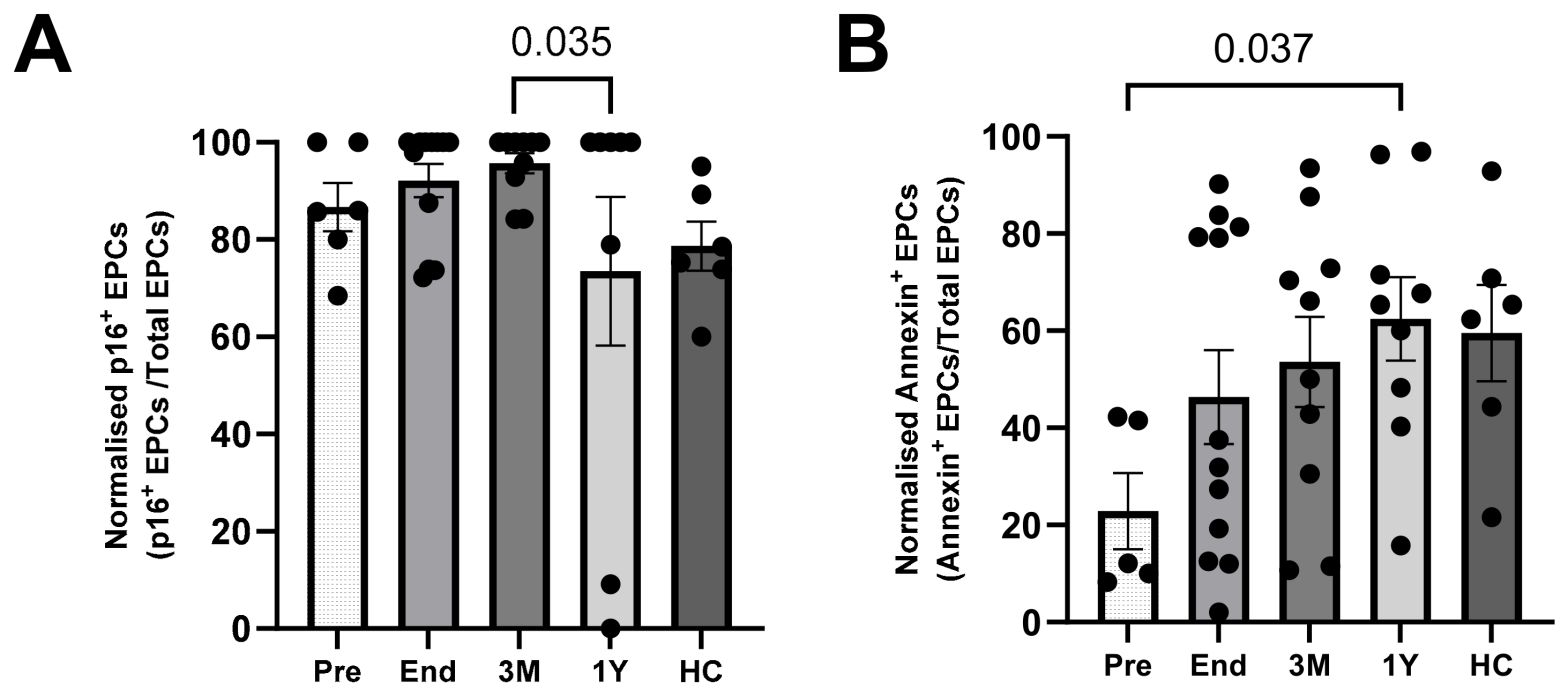
(**A**) **Senescent EPC enumeration in lymphoma patients at pre-treatment (*n* = 6), end treatment (*n* = 12), 3 months post-treatment (*n* = 10), 1 year post-treatment (*n* = 8), and healthy controls (*n* = 6).** Percentage of EPCs positive for a p16 signal relative to total EPCs. Due to non-normal residuals in both raw-scale models and the presence of zero values, an ordered beta mixed-effects model was used to determine timepoint effect. Pairwise comparisons, including HCs, were adjusted for multiple testing using the Holm–Šídák method. (**B**) **Apoptotic EPC enumeration in lymphoma patients at pre-treatment (*n* = 5), end treatment (*n* = 12), 3 months post-treatment (*n* = 10), 1 year post-treatment (*n* = 9), and healthy controls (*n* = 6).** Percentage of EPCs with a positive Annexin V signal relative to total EPCs. As both raw-scale models showed no significant deviations from residual normality, a linear mixed-effects model with a patient random intercept was used. Pairwise comparisons, including HCs, were adjusted for multiple testing using the Tukey method. Data are presented as a scatterplot of mean ± SEM. Numerical values and raw data are presented in the Supplementary Materials (Table S2 and Excel).

In this study, the senescent p16^+^ EPCs are generally in the range between 73% and 95% of all the EPCs detected. p16^+^ EPCs were elevated in lymphoma participants at End and 3 months post-treatment relative to healthy controls (similar trend observed in leukaemia patients), suggesting that the cytotoxic nature of anthracyclines triggered the senescence pathway in EPCs (Figure 5A). An ordered beta mixed-effects model with timepoint as a fixed effect and patient as a random intercept was used, and there was no statistically significant overall effect of timepoint (likelihood-ratio χ^2^(3) = 7.08, *p* = 0.069).

The apoptotic Annexin V^+^ cells were in the range between 23% and 62% of all the EPCs detected. The proportion of apoptotic EPCs was lower than in healthy controls at pre-treatment (Figure 5B). While this observation is not established, this was expected, as EPCs are required to vascularise and support lymphoma tumours [45]. Apoptotic EPCs gradually increased until 1 year post-treatment to levels comparable to healthy controls. A raw-scale linear mixed-effects model with a patient random intercept was used and a statistically significant overall timepoint effect (F(3,21) = 4.58, *p* = 0.0128) was found.

Altogether, these data demonstrate that the proportion of senescent EPCs deviates from healthy levels and reiterates that endothelial impairment during remission is likely due to defective repair mechanisms.

### 3.7. Cardiac Protein Biomarkers in Lymphoma Patients

**Figure 6:**
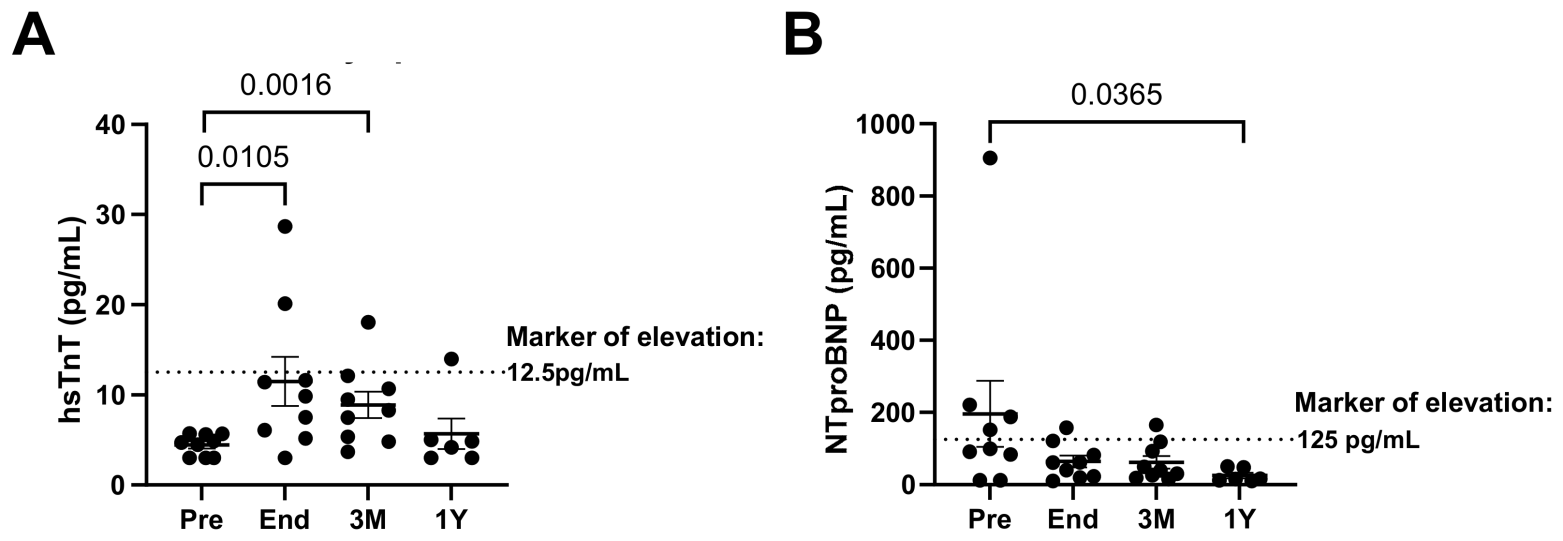
**Assay of cardiac biomarkers in lymphoma patients at pre-treatment (*n* = 9), end treatment (*n* = 9), 3 months post-treatment (*n* = 9), and 1 year post-treatment (*n* = 6).** (**A**) Assay of high-sensitivity troponin T (hsTnT). Data were log-transformed due to non-normal residuals in both raw-scale models. The heterogeneous-variance linear mixed-effects model was selected based on improved residual normality (*p* = 0.11) and lower AIC. Pairwise comparisons were adjusted for multiple testing using the Holm–Šídák method. (**B**) Assay of NTproBNP. Data were log-transformed due to non-normal residuals in both raw-scale models and subsequently analysed using a linear mixed-effects model. Pairwise comparisons were adjusted for multiple testing using the Holm–Šídák method. Data are presented as a scatterplot of mean ± SEM. Numerical values and raw data are presented in the Supplementary Materials (Excel).

All patients had normal hsTnT levels before cancer treatment (4.45 ± 0.39 pg/mL). Although hsTnT significantly increased at end treatment (11.5 ± 2.7 pg/mL), indicating significant cardiomyocyte damage, only two participants had levels above the cutoff of elevation. The levels decreased from End to 3 months post-treatment (8.88 ± 1.5 pg/mL), with all except one participant returning to below the cutoff. By 1 year post-treatment, the hsTnT levels of almost all participants returned to normal levels (5.67 ± 1.7 pg/mL) (Figure 6A). Collectively, the data suggest marginal cardiomyocyte damage occurred in lymphoma participants at the end timepoint. The linear mixed-effects model, with timepoint as a fixed effect and patient as a random intercept, showed a statistically significant overall timepoint effect (F(3,21) = 10.25, *p* = 0.0002).

Additionally, NTproBNP levels of most participants after treatment were in the acceptable range, implying that there was no significant cardiac remodelling (Figure 6B). This finding is supported by the preservation of LVEF within the normal range. The log-transformed linear mixed-effects model, with timepoint as a fixed effect and patient as a random intercept, showed a borderline statistically significant overall timepoint effect (F(3,21) = 3.14, *p* = 0.047). The Spearman test was performed to identify the correlation between hsTnT (cardiomyocyte damage) and CECs (endothelial damage) and hsTnT and EPCs, respectively. However, there were no significant correlations revealed (Figure S3). This could be due to the lack of significant cardiomyocyte damage in the lymphoma population of this study.

## 4. Discussion

This study provides a longitudinal account of the endothelial health of children with leukaemia and lymphoma, by comprehensively reporting the proportion of circulating ECs and EPCs in the peripheral blood and assessing the fate of EPCs (i.e., apoptotic or senescence), up till 1 year post-treatment. In both leukaemia and lymphoma, CECs serve as markers of endothelial damage, while EPCs are associated not only with endothelial repair but also with angiogenesis and cancer progression. Therefore, their interpretation requires careful consideration. The proportions of CECs, EPCs, and their characteristics at the pre-treatment stage are largely influenced by the underlying malignancy. In contrast, as participants were confirmed to be in remission from the end-of-treatment onwards, alterations from then on were presumed to arise mainly from the residual effects of anthracycline exposure, with negligible contribution from ongoing malignancy.

The data demonstrate that endothelial injury (represented by CECs) resolves by treatment completion in childhood cancer survivors who received anthracycline treatment. However, the persistent decline in EPCs during remission identifies defective endothelial regeneration. The dominance of senescent cells within the EPC pool during remission, in addition to lower EPC levels, further supports this. Collectively, these data suggest that long-term cardiovascular vulnerability of survivors stems from an exhausted regenerative reservoir.

It is established that anthracyclines represent a double-edged sword. That is, while they are highly effective anticancer agents, their use is limited by the risk of acute and chronic cardiovascular toxicity. For example, cardiotoxicity represents a particularly prevalent complication, where up to 57% of children who have been treated with anthracyclines exhibit cardiac abnormalities [46], and up to 16% develop congestive heart failure [47]. Additionally, late-onset cardiotoxicity, which occurs years after treatment completion, is exhibited via impaired myocardial growth and function [47,48].

Since the 1970s, research on anthracycline-induced cardiotoxicity has predominantly focused on cardiomyocytes. Given that the muscle-to-capillary ratio is 1:1 [3,49] and the proximity between capillary endothelial cells and cardiomyocytes high (i.e., 1 µm), it is realistic that anthracycline-related cardiac dysfunction is caused not only by impaired cardiomyocytes but also facilitated by compromised endothelial cells [3]. Hence, over the past decade, increasing attention has been directed towards the role of the vascular endothelium in mediating anthracycline-induced cardiac dysfunction.

Anthracycline-induced endotheliotoxicity has been demonstrated in various models. Wilkinson et al. demonstrated a reduction in the expression of the tight junction protein zona occludens in doxorubicin-treated human cardiac microvascular endothelial cells, resulting in an increase in drug permeability [3]. Treatment of EA.hy926 cell line, a combination of HUVECs and A549 human lung carcinoma cell line, with anthracyclines exerting cytotoxic effects on the nuclear and cytosolic front. [50]. Anthracyclines were accumulated in the nucleus in those cells, even with treatment at nanomolar concentrations [50]. In addition, an increase in reactive oxygen species and reorganization of the F-actin cytoskeleton were observed [50]. Another study on doxorubicin-treated HUVECs showed that the protein C anticoagulant pathway is downregulated, promoting a prothrombotic phenotype [51]. A doxorubicin-treated mouse model revealed greater accumulation of doxorubicin in endothelial nuclei than in other cardiac cell types [52]. Pigs treated with a cumulative dose of 2.25 mg/kg doxorubicin displayed structural damage to the arteries based on histological and myograph evaluations [53]. In children, vascular endothelial dysfunction was assessed using the brachial artery reactivity test in those who had completed anthracycline treatment at least two months prior [54]. However, their cardiac function was not reported. Altogether, these findings show that anthracyclines exert molecular, structural, and functional changes in endothelial cells resulting in detrimental effects from the cellular-to-organ systemic levels in various organisms. However, the condition of the endothelium at a later timepoint post-treatment has not been well-characterized [8,55,56].

### 4.1. Endothelial Health in Leukaemic Patients

This study showed that, in leukaemia patients, significant endothelial injury appeared to occur primarily during active malignancy, which normalized during remission. However, EPC counts remain lower than in healthy controls, suggesting impaired endothelial repair capacity during remission. The finding that CEC [57] and EPC counts are highest at initial diagnosis is consistent with another study conducted on adult AML patients [20].

This study revealed persistent senescent EPCs during the remission period. Although there are a lack of data on the fraction of senescent EPCs in leukaemia patients treated with anthracyclines, a large proportion of senescent cells is expected due to the inflamed environment caused by leukaemia and anthracycline treatment [58,59]. This is because inflammatory molecules activate the retinoblastoma pathway, triggering p16 expression, and inducing irreversible cell cycle arrest [9,58,59]. The data suggest that while cancer-driven endothelial injury resolves with treatment, the persistence of senescent EPCs, especially amongst a lower absolute pool, may hinder vascular recovery, predisposing survivors to long-term vascular dysfunction and cardiovascular complications. The impact of anthracyclines on apoptotic circulating EPCs has not been investigated in leukaemia patients before. However, a previous study showed that approximately 40% of precultured primary bone marrow EPCs from adult AML patients were apoptotic before treatment [60]. While it may seem like the proportion of apoptotic EPCs in healthy controls is high, no studies have reported this before, and the findings from this study cannot be directly compared with existing data. The result may be explained by an intrinsic anti-tumour surveillance mechanism, although further studies are required to substantiate this hypothesis.

Cardiac injury based on the hsTnT assay revealed significant cardiomyocyte injury at End and 3 months post-treatment. This is corroborated by a similar study on paediatric leukaemia patients treated with anthracyclines, where hsTnT levels were significantly elevated at 1 week post-treatment (11.53 pg/mL) and 6 months post-treatment (4.87 pg/mL) when compared to the pre-treatment timepoint (3.50 pg/mL) [61]. The data from this study and those of others demonstrate that delayed cardiomyocyte injury takes place in anthracycline-treated leukaemia patients. However, when hsTnT concentrations were correlated with CEC counts to assess the relationship between cardiomyocyte and endothelial injury, no significant association was observed. In future studies, the use of cardiac-specific endothelial injury markers could enhance experimental robustness.

Overall, there is evidence that endothelial injury is resolved by treatment completion, while reparative capacity remains compromised in leukaemia patients. This unresolved impairment could be an important contributor of later vascular complications.

### 4.2. Endothelial Health in Lymphoma Patients

Just as in leukemic patients, elevated CECs may arise from either stress-induced endothelial injury or cancer-driven angiogenesis, while increased EPCs can indicate endothelial repair or cancer-associated angiogenesis [28,62]. Since participants were in remission from the end-treatment timepoint onwards, subsequent changes were not attributed to cancer progression. This study shows that lymphoma participants experience a significant increase in CECs during active malignancy. This is supported by the fact that lymphoma and other cells in the microenvironment can promote angiogenesis, which involves the disruption of existing blood vessels, resulting in CECs [63]. Alongside this, endothelial injury could be caused by the inflamed environment. Nevertheless, CEC levels normalized during remission, suggesting that vascular injury had subsided. Although the counts were statistically comparable to those of healthy controls, a gradual upwards trend was observed from three months post-treatment onwards, suggesting the beginning of late-endothelial cytotoxicity. This is plausible as a follow-up study on survivors of adolescent Hodgkin Lymphoma showed that the CEC proportion was significantly elevated even after approximately 12 years from diagnosis [64]. As for EPCs, it is no surprise that they are highest during malignancy [29,45]. While the decrease in EPCs thereafter implies remission of lymphoma and a positive response to chemotherapy [45,65], the counts decreasing below healthy levels suggests that endothelial repair is impaired.

To date, no studies have characterized EPC fate in paediatric lymphoma. This study shows that the proportion of senescent EPCs in lymphoma patients is higher than in healthy controls, likely reflecting the pro-inflammatory environment induced by both the malignancy and anthracycline treatment [59,66,67,68]. Our data show that the proportion of apoptotic EPCs increases gradually. It is possible that the levels eventually exceed those of healthy controls, as impaired EPC function has been reported in adult lymphoma patients who remained in complete remission for at least two years [69]. Lymphoma participants also exhibited the most cardiomyocyte damage at the end timepoint, which largely resolved by 3 months post-treatment. However, no significant correlation was observed between hsTnT concentrations and CEC counts.

Altogether, this study demonstrates that impairment of vascular repair mechanisms is more profound and persistent than direct vascular injury itself. This suggests that defective endothelial regeneration and persistent dysfunction in the endothelial progeny of damaged progenitor cells are potential contributors of long-term vascular complications potentially leading to late-onset cardiac complications in cancer survivors [66]. This and other studies also demonstrate that apoptosis is no longer the principal mechanism underlying dox-induced endothelial injury [42].

These findings highlight the need for therapeutic strategies aimed at restoring EPC numbers and function following anthracycline treatment. The importance of EPCs in vascular health is well-supported by research showing that higher EPC levels are associated with a reduced risk of cardiovascular disease. EPCs can be further differentiated into “early” and “late” cells based on their functions. It is shown that early EPCs protect themselves from apoptosis under oxidative stress [70,71], such as anthracycline treatment through paracrine factors. While this paper primarily discusses protection against apoptosis, the interconnected nature of signalling pathways in injured cells suggests that early EPCs may also protect themselves from senescence. A related study on rats with doxorubicin-induced cardiomyopathy showed that pre-treatment with erythropoietin reduced cardiac injury [6]. Incidentally, mice treated with erythropoietin exhibited higher EPC counts and increased myocardial vascular density, postulating that EPCs may mediate these protective effects. Moreover, at least 150 interventional clinical trials involving EPCs have been registered, highlighting the growing significance in a broad spectrum of conditions, such as ischemic and metabolic diseases [72,73,74]. Collectively, these studies strongly demonstrate the potential of preserving EPC health as a preventive strategy against late-onset cardiac dysfunction following anthracycline treatment.

### 4.3. Limitations

While this study provides important insights, several limitations that require certain assumptions during the analysis should be acknowledged. We were unable to determine whether CECs originated specifically from cardiac vasculature. Therefore, although systemic fluctuations in CECs may potentially reflect concurrent alterations in the cardiac endothelium, this remains speculative and cannot be established from the present findings. To address this limitation, future work should focus on developing cardiac endothelium-specific markers [75]. It remains challenging to distinguish whether the EPCs are assigned for vascular repair or cancer-driven angiogenesis. Techniques such as flow cytometry, cell surface biotinylation, and mass spectrometry-based proteomics could help identify EPC subsets with unique surface markers characteristic of each process. As with most cancer patients, participants in this study were administered additional medications that may have influenced CEC and EPC counts [54]. As CEC and EPC levels were expressed relative to the PBMC population, treatment-related changes in PBMC composition, including changes in circulating lymphocyte populations, may have influenced the measured frequencies. Nonetheless, given the standardized treatment regimens within each type of cancer, inter-individual variability is expected to be minimal. It was assumed that the effect of cancer is so minimal from the end timepoint onward, and thus unlikely to significantly affect the CEC and EPC counts. Owing to the limited number of cells available in some buffy coat samples, a total of 10,000 PBMC events could not be acquired by flow cytometry. Therefore, calculations were performed using the total number of available PBMC events for each sample. The raw counts are provided as Supplementary Materials. Also, cell enumeration experiments were performed first, followed by EPC characterisation experiments, by which time some of the stored samples from individual participants had been exhausted. Therefore, EPC characterisation was performed using the remaining available samples, resulting in the differences in sample size.

## 5. Conclusions

This study demonstrates that impaired endothelial repair during cancer remission, particularly driven by the reduced quantity and functional quality of EPCs, may underlie the increased risk of long-term cardiovascular complications in cancer survivors. Although further investigation is warranted, therapeutic and preventive strategies targeting EPC restoration and function may hold promise in mitigating the cardiovascular and socioeconomic burden faced by cancer survivors.

## Figures and Tables

**Table 1:** Demographics of research participants. There was no statistically significant difference in age between patients and healthy participants (*p* = 0.07).

	Patients (*n* = 48)	Healthy Controls (*n* = 10)
Male (%)	34 (70.8%)	7 (70%)
Participants’ age	9.4 ± 0.8	12.1 ± 1.4
Cumulative anthracycline dose (mg/m^2^)	172 ± 12.4	-
%LVEF	61.3 ± 0.4	-

**Table 2:** Clinical laboratory characteristics of research participants. #, $, *, and ^ symbols indicate statistically significant differences with Pre, End, 3M, and 1Y timepoints, respectively. Statistical analyses: Student’s t-tests and their non-parametric equivalents.

	Leukaemia				Lymphoma			
	Pre	End	3M	1Y	Pre	End	3M	1Y
**WBC** **(×10^9^/L)**	7.28 ± 2.4	4.17 ± 1.0	4.63 ± 0.9	4.45 ± 0.7	9.99 ± 2.1	7.76 ± 4.6 #	4.90 ± 0.4 #	6.14 ± 1.3
**RBC** **(×10^12^/L)**	3.24 ± 0.1	3.38 ± 0.2	3.9 ± 0.2 #,$	4.13 ± 0.2 #,$	4.67 ± 0.3	3.68 ± 0.3 #,*,^	4.97 ± 0.1	4.79 ± 0.2
**Haemoglobin (g/dL)**	9.75 ± 0.4	9.91 ± 0.4	11.9 ± 0.4 #,$,^	12.8 ± 0.4 #,$	12.3 ± 0.7	10.4 ± 0.7 #,*,^	13.6 ± 0.3	13.7 ± 0.7
**MCV (fL)**	89.1 ± 1.1	87.6 ± 1.2	89.4 ± 1.3	86.2 ± 4.4	79.0 ± 1.3	84.9 ± 1.8 #	82.3 ± 1.4 #	85.1 ± 1.8 #
**MCH (pg)**	30.2 ± 0.4	29.5 ± 0.4 *,^	30.5 ± 0.4	31.4 ± 0.7	26.5 ± 0.8	28.6 ± 0.7 #	27.4 ± 0.6	28.5 ± 0.9
**MCHC** **(g/dL)**	33.8 ± 0.3	33.4 ± 0.4	34.1 ± 0.2	34.6 ± 0.2 #,$	33.5 ± 0.6	33.6 ± 0.3	33.3 ± 0.5	33.5 ± 0.6
**Haematocrit (%)**	28.8 ± 1.1	29.4 ± 1.2	34.8 ± 1.2 #,$	37.1 ± 1.2 #,$	36.8 ± 2.0	31.1 ± 2.3 #,*,^	40.8 ± 0.7 #	40.7 ± 1.4
**Platelets (×10^9^/L)**	224.2 ± 30.6	273.4 ± 30.7	275 ± 22.4	244.8 ± 19.5	287.8 ± 38.0	217.2 ± 27.3 *	292.4 ± 30.5	281.7 ± 35.2
**MPV (fL)**	9.78 ± 0.2	10.4 ± 0.2 #,*	9.80 ± 0.2	14.2 ± 4.5	9.74 ± 0.3	10.6 ± 0.3 #,*,^	9.85 ± 0.3	9.70 ± 0.3
**RDW (%)**	16.9 ± 0.5	16.6 ± 0.6 ^	15.7 ± 0.5 #,^	14.3 ± 0.5 #	14.3 ± 0.7	16.3 ± 1.0 #,*,^	14.0 ± 0.4	13.1 ± 0.4
**Neutrophils** **(×10^9^/L)**	1.30 ± 0.2	2.71 ± 1.0	3.39 ± 0.9 #,$	2.40 ± 0.5 #	7.37 ± 2.0	2.03 ± 0.7 #	3.00 ± 0.3 #	3.47 ± 0.8 #
**Lymphocytes** **(×10^9^/L)**	1.27 ± 0.2	0.97 ± 0.09	0.68 ± 0.06 #,$,^	1.43 ± 0.26	1.73 ± 0.31	0.93 ± 0.18 #	1.23 ± 0.16	1.97 ± 0.51
**Neutrophils/** **Lymphocytes**	1.95 ± 0.38	2.98 ± 0.98	5.70 ± 1.4 #,$,^	2.08 ± 0.26	7.35 ± 3.1	2.03 ± 0.47	3.01 ± 0.73	1.76 ± 0.28
**Monocytes** **(×10^9^/L)**	0.501 ± 0.2	0.487 ± 0.05 #	0.415 ± 0.05	0.422 ± 0.05	0.635 ± 0.1	0.397 ± 0.06	0.493 ± 0.06	0.493 ± 0.06
**Eosinophils** **(×10^9^/L)**	0.0922 ± 0.03	0.0489 ± 0.02 *,^	0.117 ± 0.03	0.168 ± 0.04 #	0.210 ± 0.09	0.0556 ± 0.02 *,^	0.155 ± 0.02	0.165 ± 0.04
**Basophils (×10^9^/L)**	0.0107 ± 0.003	0.0193 ± 0.003 #	0.0236 ± 0.004 #	0.0275 ± 0.005 #	0.0327 ± 0.007	0.0167 ± 0.003 *,^	0.0290 ± 0.005	0.0433 ± 0.01

## Data Availability

The data presented in this study are available on request from the corresponding authors.

## Supplementary Materials

The following supporting information can be downloaded at: https://www.mdpi.com/article/10.3390/cells15212040/s1.

## Abbreviations

The following abbreviations are used in this manuscript:

ALL	Acute lymphoblastic leukaemia
AML	Acute myeloid leukaemia
BNP	Brain natriuretic peptide
BSA	Bovine serum albumin
CECs	Circulating endothelial cells
EPCs	Endothelial progenitor cells
HsTnT	High-sensitivity cardiac troponin T
LVEF	Left ventricular ejection fraction
NLR	Neutrophil-to-lymphocyte ratio
NT-proBNP	N-terminal pro B-type natriuretic peptide
PBS	Phosphate-buffered saline
TnT	Troponin T
PBMC	Peripheral blood mononuclear cell
