# Supplementary material for: Compromised Endothelial Regeneration as a Potential Contributor to Long-Term Cardiovascular Complications in Childhood Cancer Survivors
Source: Cells. 2026 Nov 9;15(21):2040. doi: 10.3390/cells15212040 (PMC13761598; doi:10.3390/cells15212040)
Supplement: Supplementary file 1 [file cells-15-02040-s001.zip › Supplemental Appendix for cells - Statistics (Supplementary S3).pdf]

%CEC – Leukaemia (Without HC)

```
> # Shapiro-Wilk test for equal-variance model
> shapiro.test(resid_raw_equal)
Shapiro-Wilk normality test
data:  resid_raw_equal
W = 0.93893, p-value = 0.0004343

> # Shapiro-Wilk test for heterogeneous-variance model
> shapiro.test(resid_raw_hetero)
Shapiro-Wilk normality test
data:  resid_raw_hetero
W = 0.87489, p-value = 4.565e-07
```

Conclusion: Shapiro–Wilk tests indicated departures from normality in the normalized residuals of both the homogeneous-variance model.

After log-transformation,

```
> anova(model_log_equal, model_log_hetero)
                 Model df      AIC      BIC    logLik   Test  L.Ratio p-value
model_log_equal      1  6 318.1037 332.6886 -153.0519
model_log_hetero     2  9 321.0968 342.9742 -151.5484 1 vs 2 3.006875  0.3906

  > shapiro.test(resid_log_equal)
  Shapiro-Wilk normality test
  data:  resid_log_equal
  W = 0.97426, p-value = 0.07677

  > shapiro.test(resid_log_hetero)
  Shapiro-Wilk normality test
  data:  resid_log_hetero
  W = 0.97932, p-value = 0.1724
```

Conclusion: model_log_equal is favourable.

```
  >  final_model <- model_log_equal
  > # Overall timepoint effect
  > anova(final_model, type = "marginal")
              numDF denDF  F-value p-value
  (Intercept)     1    54 3.967849  0.0514
  Timepoint       3    54 4.310806  0.0085
```

Conclusion: yes, there is significant difference across the timepoints.

%CEC – Leukaemia (HC)

```
> # Shapiro-Wilk test for equal-variance model
> shapiro.test(resid_raw_equal)
Shapiro-Wilk normality test
data:  resid_raw_equal
W = 0.92182, p-value = 2.779e-05

> # Shapiro-Wilk test for heterogeneous-variance model
> shapiro.test(resid_raw_hetero)
Shapiro-Wilk normality test
data:  resid_raw_hetero
W = 0.81002, p-value = 1.018e-09
```

Conclusion: Shapiro–Wilk tests indicated departures from normality in the normalized residuals of both the homogeneous-variance model.

After log-transformation,

```
> # Compare variance structures within the log-scale models
> anova(model_log_equal, model_log_hetero)
                 Model df      AIC      BIC    logLik   Test L.Ratio p-value
model_log_equal      1  7 339.4894 356.9880 -162.7447
model_log_hetero     2 11 341.9058 369.4037 -159.9529 1 vs 2 5.58357  0.2325

> shapiro.test(resid_log_equal)
Shapiro-Wilk normality test
data:  resid_log_equal
W = 0.97738, p-value = 0.09901

> shapiro.test(resid_log_hetero)
Shapiro-Wilk normality test
data:  resid_log_hetero
```

W = 0.98236, p-value = 0.2303

Conclusion: model_log_equal is favourable.

```
> print(results)
```

contrast	ratio	SE	df	null	t.ratio	p.value	p_holm_sidak	significant_0.05
Pre / End	2.773773	0.9803515	54	1	2.887	0.0056	0.0491961	TRUE
Pre / 3M	3.439238	1.2613414	54	1	3.368	0.0014	0.0139295	TRUE
Pre / 1Y	1.895134	0.7108773	54	1	1.704	0.0941	0.5463341	FALSE
Pre / HC	2.165193	1.3180666	36	1	1.269	0.2126	0.7616430	FALSE
End / 3M	1.239913	0.4469171	54	1	0.597	0.5533	0.9140492	FALSE
End / 1Y	0.683233	0.2546253	54	1	-1.022	0.3113	0.8450463	FALSE
End / HC	0.780595	0.4754301	36	1	-0.407	0.6866	0.9140492	FALSE
3M / 1Y	0.551033	0.2098547	54	1	-1.565	0.1235	0.6024309	FALSE
3M / HC	0.629556	0.3887909	36	1	-0.749	0.4585	0.9140492	FALSE
1Y / HC	1.142501	0.7133635	36	1	0.213	0.8322	0.9140492	FALSE

%EPC – Leukaemia

```
> shapiro.test(resid_raw_equal)
Shapiro-Wilk normality test
data:  resid_raw_equal
W = 0.7949, p-value = 3.54e-10

> # Shapiro-Wilk test for heterogeneous-variance model
> shapiro.test(resid_raw_hetero)
Shapiro-Wilk normality test
data:  resid_raw_hetero
W = 0.7538, p-value = 2.544e-11
```

Conclusion: Shapiro–Wilk tests indicated departures from normality in the normalized residuals of both the homogeneous-variance model.

After log-transformation,

```
> anova(model_log_equal, model_log_hetero)
                 Model df      AIC      BIC    logLik   Test  L.Ratio p-value
model_log_equal      1  6 327.5312 342.5964 -157.7656
model_log_hetero     2  9 325.5297 348.1275 -153.7649 1 vs 2 8.001472   0.046

> shapiro.test(resid_log_equal)
Shapiro-Wilk normality test
data:  resid_log_equal
W = 0.98106, p-value = 0.1855

> shapiro.test(resid_log_hetero)
Shapiro-Wilk normality test
data:  resid_log_hetero
W = 0.97626, p-value = 0.08156
```

Conclusion: model_log_hetero is favourable.

```
  >   final_model <- model_log_hetero
  > # Overall timepoint effect
  > anova(final_model, type = "marginal")
            numDF denDF  F-value p-value
  (Intercept)     1    58 1.807096  0.1841
  Timepoint       3    58 2.490490  0.0692
```

Conclusion: There is no significant difference across the timepoints.

%EPC – Leukaemia (HC)

```
> shapiro.test(resid_raw_equal)
Shapiro-Wilk normality test
data:  resid_raw_equal
W = 0.79955, p-value = 1.815e-10

> # Shapiro-Wilk test for heterogeneous-variance model
> shapiro.test(resid_raw_hetero)
Shapiro-Wilk normality test
data:  resid_raw_hetero
W = 0.76961, p-value = 2.38e-11
```

Conclusion: Shapiro–Wilk tests indicated departures from normality in the normalized residuals.

After log-transformation,

anova(model_log_equal, model_log_hetero)

	Model	df	AIC	BIC	logLik	Test	L.Ratio	p-value
model_log_equal	1	7	346.0649	364.0879	-166.0324			
model_log_hetero	2	11	338.8799	367.2018	-158.4400	1 vs 2	15.18493	0.0043

> shapiro.test(resid_log_equal)
Shapiro-Wilk normality test
data: resid_log_equal
W = 0.98282, p-value = 0.208

> shapiro.test(resid_log_hetero)
Shapiro-Wilk normality test
data: resid_log_hetero
W = 0.98001, p-value = 0.1252
Conclusion: model_log_hetero is favourable.

> final_model <- model_log_hetero

> results$p_holm_sidak <- holm_sidak(results$p.value)
> results$significant_0.05 <- results$p_holm_sidak < 0.05
> print(results)

contrast	ratio	SE	df	null	t.ratio	p.value	p_holm_sidak	significant_0.05
Pre / End	0.8975100	0.3189611	58	1	-0.304	0.7620	0.9286856	FALSE
Pre / 3M	1.5511971	0.6113091	58	1	1.114	0.2699	0.6107609	FALSE
Pre / 1Y	1.7304605	0.6170365	58	1	1.538	0.1295	0.4257900	FALSE
Pre / HC	0.4466804	0.1592759	39	1	-2.260	0.0295	0.1643085	FALSE
End / 3M	1.7283342	0.5517678	58	1	1.714	0.0919	0.3824156	FALSE
End / 1Y	1.9280682	0.5264882	58	1	2.404	0.0194	0.1282715	FALSE
End / HC	0.4976885	0.1377610	39	1	-2.521	0.0159	0.1203891	FALSE
3M / 1Y	1.1155645	0.3558394	58	1	0.343	0.7330	0.9286856	FALSE
3M / HC	0.2879585	0.0933053	39	1	-3.842	0.0004	0.0039324	TRUE
1Y / HC	0.2581280	0.0720926	39	1	-4.849	<0.0001	0.0002014	TRUE

p16+EPC/EPC (%) – Leukaemia (Without HC)

> shapiro.test(resid_raw_equal)
Shapiro-Wilk normality test
data: resid_raw_equal
W = 0.9492, p-value = 0.006104

> # Shapiro-Wilk test for heterogeneous-variance model
> shapiro.test(resid_raw_hetero)
Shapiro-Wilk normality test
data: resid_raw_hetero
W = 0.92753, p-value = 0.00052

Conclusion: Both residuals are not normally distributed. But because the dataset contains zero, we also cannot do a log-transformation to model it accordingly. Hence, we will use the ordered beta mixed-effects model.

Models:
model_ordbeta_null: Proportion ~ (1 | Patient), zi=~0, disp=~1
model_ordbeta: Proportion ~ Timepoint + (1 | Patient), zi=~0, disp=~1

	Df	AIC	BIC	logLik	deviance	Chisq	Chi Df	Pr(>Chisq)
model_ordbeta_null	5	30.063	41.376	-10.0315	20.063			
model_ordbeta	8	33.846	51.947	-8.9228	17.846	2.2174	3	0.5285

Conclusion: An ordered beta mixed-effects model, with timepoint as a fixed effect and patient as a random intercept, found no statistically significant overall effect of timepoint (likelihood-ratio $\chi^2(3) = 2.22$, p = 0.529).

p16+EPC/EPC (%) – Leukaemia (HC)

> shapiro.test(resid_raw_equal)
Shapiro-Wilk normality test
data: resid_raw_equal
W = 0.9492, p-value = 0.001656

> # Shapiro-Wilk test for heterogeneous-variance model
> shapiro.test(resid_raw_hetero)
Shapiro-Wilk normality test
data: resid_raw_hetero
W = 0.92753, p-value = 0.0001481

Conclusion: Shapiro–Wilk tests indicated departures from normality in the normalized residuals of both the homogeneous-variance model.

```
> print(overall_timepoint_test)
Data: dat
Models:
model_ordbeta_null: Proportion ~ (1 | Patient), zi=~0, disp=~1
model_ordbeta: Proportion ~ Timepoint + (1 | Patient), zi=~0, disp=~1
                   Df    AIC    BIC  logLik deviance  Chisq Chi Df Pr(>Chisq)
model_ordbeta_null  5 25.869 37.588 -7.9346   15.869
model_ordbeta       9 31.394 52.488 -6.6968   13.394 2.4755      4      0.649

> print(pairwise_results)
 contrast    estimate        SE  df z.ratio p.value p_holm_sidak significant_0.05
 Pre - End -0.3791248 0.2602405 Inf  -1.457  0.1452    0.7916352 FALSE
 Pre - 3M  -0.1887842 0.2615152 Inf  -0.722  0.4704    0.9924635 FALSE
 Pre - 1Y  -0.0716280 0.2534487 Inf  -0.283  0.7775    0.9984043 FALSE
 Pre - HC  -0.2235288 0.5051975 Inf  -0.442  0.6582    0.9984043 FALSE
 End - 3M   0.1903405 0.2560137 Inf   0.743  0.4572    0.9924635 FALSE
 End - 1Y   0.3074968 0.2545315 Inf   1.208  0.2270    0.9014742 FALSE
 End - HC   0.1555960 0.5068220 Inf   0.307  0.7588    0.9984043 FALSE
 3M - 1Y    0.1171562 0.2655363 Inf   0.441  0.6591    0.9984043 FALSE
 3M - HC   -0.0347445 0.5120300 Inf  -0.068  0.9459    0.9984043 FALSE
 1Y - HC   -0.1519008 0.5035440 Inf  -0.302  0.7629    0.9984043 FALSE
Results are given on the log odds ratio (not the response) scale.
```

Conclusion: None of the 10 pairwise comparisons was statistically significant after Holm–Šídák adjustment (all adjusted p ≥ 0.792).

AnnexinV+EPC/EPC (%) – Leukaemia (without HC)

```
>Shapiro-Wilk normality test
data:  resid_raw_equal
W = 0.98082, p-value = 0.3497

> # Shapiro-Wilk test for heterogeneous-variance model
> shapiro.test(resid_raw_hetero)
Shapiro-Wilk normality test
data:  resid_raw_hetero
W = 0.98022, p-value = 0.3251
```

Conclusion: The Shapiro–Wilk test did not detect a departure from normality in the normalized residuals.

```
> anova(final_model, type = "marginal")
            numDF denDF   F-value p-value
(Intercept)     1    41 134.87987  <.0001
Timepoint       3    41   1.75093  0.1717
```

Conclusion: A raw-scale linear mixed-effects model with a patient random intercept found no statistically significant overall effect of timepoint (F(3,41) = 1.75, p = 0.172).

AnnexinV+EPC/EPC (%) – Leukaemia (HC)

```
> shapiro.test(resid_raw_equal)
Shapiro-Wilk normality test
data:  resid_raw_equal
W = 0.98084, p-value = 0.2978

> # Shapiro-Wilk test for heterogeneous-variance model
> shapiro.test(resid_raw_hetero)
Shapiro-Wilk normality test
data:  resid_raw_hetero
W = 0.97951, p-value = 0.249
```

Conclusion: The Shapiro–Wilk test did not detect a departure from normality in the normalized residuals.

```
> # Overall test: are the four timepoint means equal?
> anova(final_model, type = "marginal")
            numDF denDF   F-value p-value
(Intercept)     1    40 131.02840  <.0001
Timepoint       4    40   1.45743  0.2332

> # All six pairwise comparisons
> summary(
+   pairs(emm, adjust = "tukey"),
+   infer = c(TRUE, TRUE)
+ )
 contrast  estimate   SE df lower.CL upper.CL t.ratio p.value
```

```
 Pre - End    0.551 6.89 41   -19.12     20.2   0.080  1.0000
 Pre - 3M    13.542 6.98 41    -6.38     33.5   1.939  0.3138
 Pre - 1Y     6.976 7.08 41   -13.23     27.2   0.985  0.8606
 Pre - HC    -2.235 9.81 31   -30.62     26.2  -0.228  0.9994
 End - 3M    12.991 6.79 41    -6.39     32.4   1.912  0.3271
 End - 1Y     6.425 6.89 41   -13.24     26.1   0.932  0.8828
 End - HC    -2.786 9.67 31   -30.78     25.2  -0.288  0.9984
 3M - 1Y     -6.566 6.98 41   -26.49     13.4  -0.940  0.8795
 3M - HC    -15.777 9.74 31   -43.96     12.4  -1.621  0.4960
 1Y - HC     -9.211 9.81 31   -37.60     19.2  -0.939  0.8793
```

Conclusion: A raw-scale linear mixed-effects model, with group/visit category as a fixed effect and participant as a random intercept, found no statistically significant overall group/visit effect ($F(4,40) = 1.46$, $p = 0.233$). None of the 10 pairwise comparisons was statistically significant after Tukey adjustment (all adjusted $p \geq 0.314$). The estimated participant random-intercept variance was negligible.

(hsTnT (pg/mL)) – Leukaemia

```
> shapiro.test(resid_raw_equal)
Shapiro-Wilk normality test
data:  resid_raw_equal
W = 0.81526, p-value = 7.324e-08

> # Shapiro-Wilk test for heterogeneous-variance model
> shapiro.test(resid_raw_hetero)
Shapiro-Wilk normality test
data:  resid_raw_hetero
W = 0.80865, p-value = 4.821e-08
```

Conclusion: Shapiro–Wilk tests indicated departures from normality in the normalized residuals.

After log-transformation,

```
> anova(model_log_equal, model_log_hetero)
                 Model df      AIC      BIC    logLik   Test  L.Ratio p-value
model_log_equal      1  6 115.0019 128.0483 -51.50097
model_log_hetero     2  9 118.6339 138.2034 -50.31697 1 vs 2 2.368003  0.4996

> shapiro.test(resid_log_equal)
Shapiro-Wilk normality test
data:  resid_log_equal
W = 0.96268, p-value = 0.03742

> shapiro.test(resid_log_hetero)
Shapiro-Wilk normality test
data:  resid_log_hetero
W = 0.95194, p-value = 0.009869
```

Conclusion: Even after log-transformation, the residuals do deviate from normality. But between the two, model_log_equal is more favourable.

```
> final_model <- model_log_equal
> # Overall timepoint effect
> anova(final_model, type = "marginal")
            numDF denDF   F-value p-value
(Intercept)     1    46 172.57908  <.0001
Timepoint       3    46  20.75607  <.0001
```

Conclusion: yes, there is significant difference across the timepoints.

```
> results$p_holm_sidak <- holm_sidak(results$p.value)
> results$significant_0.05 <- results$p_holm_sidak < 0.05
> print(results)
 contrast      ratio        SE df null t.ratio p.value p_holm_sidak significant_0.05
 Pre / End 0.3914780 0.0594723 46    1  -6.173 <0.0001    0.0000008  TRUE
 Pre / 3M  0.5873506 0.0884002 46    1  -3.536  0.0009    0.0028191  TRUE
 Pre / 1Y  1.0888494 0.1669199 46    1   0.555  0.5814    0.5814048 FALSE
 End / 3M  1.5003413 0.2115140 46    1   2.878  0.0061    0.0120754  TRUE
 End / 1Y  2.7813808 0.4086837 46    1   6.962 <0.0001    0.0000001  TRUE
 3M / 1Y   1.8538320 0.2678495 46    1   4.272 <0.0001    0.0003848  TRUE
```

(NTproBNP (pg/mL)) – Leukaemia

```
> shapiro.test(resid_raw_equal)
Shapiro-Wilk normality test
data:  resid_raw_equal
W = 0.69118, p-value = 7.566e-11
```

```
> # Shapiro-Wilk test for heterogeneous-variance model
> shapiro.test(resid_raw_hetero)
Shapiro-Wilk normality test
data:  resid_raw_hetero
W = 0.7235, p-value = 3.555e-10
```

Conclusion: Shapiro–Wilk tests indicated departures from normality in the normalized residuals.

After log-transformation,

```
> anova(model_log_equal, model_log_hetero)
                 Model df      AIC      BIC    logLik   Test   L.Ratio p-value
model_log_equal      1  6 198.3080 211.4459 -93.15399
model_log_hetero     2  9 203.5749 223.2817 -92.78743 1 vs 2 0.7331135  0.8654
```

```
> shapiro.test(resid_log_equal)
Shapiro-Wilk normality test
data:  resid_log_equal
W = 0.95683, p-value = 0.01678
```

```
> shapiro.test(resid_log_hetero)
Shapiro-Wilk normality test
data:  resid_log_hetero
W = 0.95955, p-value = 0.02365
```

Conclusion: Even after log-transformation, the residuals do deviate from normality. But between the two, model_log_equal is more favourable.

```
> final_model <- model_log_equal
> # Overall timepoint effect
> anova(final_model, type = "marginal")
            numDF denDF   F-value p-value
(Intercept)     1    47 285.93153  <.0001
Timepoint       3    47   1.29445  0.2874
```

Conclusion: No, there is no significant difference across the timepoints.

```
> results$p_holm_sidak <- holm_sidak(results$p.value)
> results$significant_0.05 <- results$p_holm_sidak < 0.05
> print(results)
 contrast     ratio        SE df null t.ratio p.value p_holm_sidak significant_0.05
 Pre / End 1.286658 0.3455611 47    1   0.938  0.3528    0.7682348 FALSE
 Pre / 3M  1.394814 0.3711668 47    1   1.250  0.2173    0.7062709 FALSE
 Pre / 1Y  1.693118 0.4586580 47    1   1.944  0.0579    0.3009100 FALSE
 End / 3M  1.084060 0.2753127 47    1   0.318  0.7520    0.7682348 FALSE
 End / 1Y  1.315904 0.3491701 47    1   1.035  0.3062    0.7682348 FALSE
 3M / 1Y   1.213866 0.3164932 47    1   0.743  0.4610    0.7682348 FALSE
```

%CEC – Lymphoma (Without HC)

```
> shapiro.test(resid_raw_equal)
Shapiro-Wilk normality test
data:  resid_raw_equal
W = 0.8432, p-value = 4.253e-05

> # Shapiro-Wilk test for heterogeneous-variance model
> shapiro.test(resid_raw_hetero)
Shapiro-Wilk normality test
data:  resid_raw_hetero
W = 0.78342, p-value = 2e-06
```

Conclusion: Shapiro–Wilk tests indicated departures from normality in the normalized residuals.

After log-transformation,

```
> anova(model_log_equal, model_log_hetero)
                Model df      AIC      BIC    logLik   Test   L.Ratio p-value
model_log_equal      1  6 155.5723 165.3978 -71.78616
model_log_hetero     2  9 160.6273 175.3656 -71.31364 1 vs 2 0.9450438  0.8145

> shapiro.test(resid_log_equal)
Shapiro-Wilk normality test
data:  resid_log_equal
W = 0.98348, p-value = 0.7938

> shapiro.test(resid_log_hetero)
Shapiro-Wilk normality test
data:  resid_log_hetero
W = 0.97609, p-value = 0.5159
```

Conclusion: model_log_equal is favourable.

```
> anova(final_model, type = "marginal")
            numDF denDF   F-value p-value
(Intercept)     1    26 0.6909424  0.4134
Timepoint       3    26 3.0308173  0.0472
```

Conclusion: yes, there is significant difference across the timepoints.

%CEC – Lymphoma (HC)

```
> shapiro.test(resid_raw_equal)
Shapiro-Wilk normality test
data:  resid_raw_equal
W = 0.8303, p-value = 4.722e-06

> # Shapiro-Wilk test for heterogeneous-variance model
> shapiro.test(resid_raw_hetero)
Shapiro-Wilk normality test
data:  resid_raw_hetero
W = 0.80464, p-value = 1.123e-06
```

Conclusion: Shapiro–Wilk tests indicated departures from normality in the normalized residuals.

After log-transformation,

```
> anova(model_log_equal, model_log_hetero)
                Model df      AIC      BIC    logLik   Test  L.Ratio p-value
model_log_equal      1  7 179.4689 192.1156 -82.73446
model_log_hetero     2 11 183.2692 203.1425 -80.63459 1 vs 2 4.199747  0.3796

> shapiro.test(resid_log_equal)
Shapiro-Wilk normality test
data:  resid_log_equal
W = 0.98461, p-value = 0.7546

> shapiro.test(resid_log_hetero)

Shapiro-Wilk normality test
data:  resid_log_hetero
W = 0.9832, p-value = 0.6922
```

Conclusion: model_log_equal is favourable.

```
> anova(final_model, type = "marginal")
numDF denDF   F-value p-value
(Intercept)     1    25 0.7792288  0.3858
```

```
Timepoint       4    25 2.5994965  0.0605

> results$p_holm_sidak <- holm_sidak(results$p.value)
> results$significant_0.05 <- results$p_holm_sidak < 0.05
> print(results)
 contrast     ratio        SE df null t.ratio p.value p_holm_sidak significant_0.05
 Pre / End 5.113856 2.6515814 26    1   3.147  0.0041    0.0402823  TRUE
 Pre / 3M  2.424500 1.2571256 26    1   1.708  0.0995    0.5921941 FALSE
 Pre / 1Y  1.879328 1.0699700 26    1   1.108  0.2779    0.8037247 FALSE
 Pre / HC  2.911328 1.8066728 19    1   1.722  0.1013    0.5921941 FALSE
 End / 3M  0.474104 0.2501775 26    1  -1.414  0.1691    0.6709856 FALSE
 End / 1Y  0.367497 0.2122210 26    1  -1.733  0.0949    0.5921941 FALSE
 End / HC  0.569302 0.3594303 19    1  -0.892  0.3834    0.8554550 FALSE
 3M / 1Y   0.775140 0.4476252 26    1  -0.441  0.6628    0.8930089 FALSE
 3M / HC   1.200795 0.7581254 19    1   0.290  0.7751    0.8930089 FALSE
 1Y / HC   1.549133 1.0477190 19    1   0.647  0.5253    0.8930089 FALSE
```

%EPC – Lymphoma (Without HC)

```
> shapiro.test(resid_raw_equal)
Shapiro-Wilk normality test
data:  resid_raw_equal
W = 0.78338, p-value = 2.487e-06

> # Shapiro-Wilk test for heterogeneous-variance model
> shapiro.test(resid_raw_hetero)
Shapiro-Wilk normality test
data:  resid_raw_hetero
W = 0.92526, p-value = 0.01008
```

Conclusion: Shapiro–Wilk tests indicated departures from normality in the normalized residuals.

After log-transformation,

```
> anova(model_log_equal, model_log_hetero)
                 Model df      AIC      BIC    logLik   Test L.Ratio p-value
model_log_equal      1  6 130.5115 140.1770 -59.25573
model_log_hetero     2  9 127.5322 142.0304 -54.76609 1 vs 2 8.97929  0.0296

> shapiro.test(resid_log_equal)
Shapiro-Wilk normality test
data:  resid_log_equal
W = 0.96974, p-value = 0.3381

> shapiro.test(resid_log_hetero)
Shapiro-Wilk normality test
data:  resid_log_hetero
W = 0.95713, p-value = 0.1247
```

Conclusion: model_log_hetero is favourable.

```
>   final_model <- model_log_hetero
> # Overall timepoint effect
> anova(final_model, type = "marginal")
            numDF denDF  F-value p-value
(Intercept)     1    25 0.424142  0.5208
Timepoint       3    25 4.829113  0.0087
```

Conclusion: yes, there is significant difference across the timepoints.

%EPC – Lymphoma (HC)

```
> shapiro.test(resid_raw_equal)
Shapiro-Wilk normality test
data:  resid_raw_equal
W = 0.79427, p-value = 7.911e-07

> # Shapiro-Wilk test for heterogeneous-variance model
> shapiro.test(resid_raw_hetero)
Shapiro-Wilk normality test
data:  resid_raw_hetero
W = 0.92308, p-value = 0.003416
```

Conclusion: Shapiro–Wilk tests indicated departures from normality in the normalized residuals.

After log-transformation,

```
> anova(model_log_equal, model_log_hetero)
                 Model df      AIC      BIC    logLik   Test  L.Ratio p-value
model_log_equal      1  7 148.8704 161.3597 -67.43519
```

```
model_log_hetero     2 11 141.2226 160.8487 -59.61132 1 vs 2 15.64774  0.0035
> shapiro.test(resid_log_equal)
Shapiro-Wilk normality test
data:  resid_log_equal
W = 0.97661, p-value = 0.4328

> shapiro.test(resid_log_hetero)
Shapiro-Wilk normality test
data:  resid_log_hetero
W = 0.96866, p-value = 0.214
```

Conclusion: model_log_hetero is favourable.

```
> results$p_holm_sidak <- holm_sidak(results$p.value)
> results$significant_0.05 <- results$p_holm_sidak < 0.05
> print(results)
 contrast     ratio       SE df null t.ratio p.value p_holm_sidak significant_0.05
 Pre / End 7.269813 3.826611 25    1   3.769  0.0009    0.0062493  TRUE
 Pre / 3M  3.919344 1.887015 25    1   2.837  0.0089    0.0437142  TRUE
 Pre / 1Y  3.991325 1.677169 25    1   3.294  0.0029    0.0175646  TRUE
 Pre / HC  0.921270 0.420112 19    1  -0.180  0.8592    0.9801740 FALSE
 End / 3M  0.539126 0.219760 25    1  -1.516  0.1422    0.3687151 FALSE
 End / 1Y  0.549027 0.184562 25    1  -1.784  0.0866    0.3040068 FALSE
 End / HC  0.126725 0.048283 19    1  -5.422 <0.0001    0.0002813  TRUE
 3M / 1Y   1.018366 0.264537 25    1   0.070  0.9447    0.9801740 FALSE
 3M / HC   0.235057 0.074279 19    1  -4.582  0.0002    0.0016277  TRUE
 1Y / HC   0.230818 0.051041 19    1  -6.630 <0.0001    0.0000242  TRUE
```

p16+EPC/EPC (%) – Lymphoma (Without HC)

```
> # Shapiro-Wilk test for equal-variance model
> shapiro.test(resid_raw_equal)
Shapiro-Wilk normality test
data:  resid_raw_equal
W = 0.78125, p-value = 7.058e-06

> # Shapiro-Wilk test for heterogeneous-variance model
> shapiro.test(resid_raw_hetero)
Shapiro-Wilk normality test
data:  resid_raw_hetero
W = 0.87276, p-value = 0.0002426
```

Conclusion: Shapiro–Wilk tests indicated departures from normality in the normalized residuals.
But attempting to model it to model_raw_hetro (Gaussian linear mixed-effects models (LMMs)) was not possible. It yields errors. Hence, we proceeded with ordered beta mixed-effects model.

```
> print(overall_timepoint_test)
Data: dat
Models:
model_ordbeta_null: Proportion ~ (1 | Patient), zi=~0, disp=~1
model_ordbeta: Proportion ~ Timepoint + (1 | Patient), zi=~0, disp=~1
                   Df    AIC    BIC  logLik deviance  Chisq Chi Df Pr(>Chisq)
model_ordbeta_null  5 53.430 61.347 -21.715   43.430
model_ordbeta       8 52.351 65.019 -18.175   36.351 7.0787      3    0.06943
```

Conclusion: There is no significant difference across the timepoints.

p16+EPC/EPC (%) – Lymphoma (without HC). The equal-residual-variance Gaussian mixed model showed evidence of departures from residual normality (Shapiro–Wilk W = 0.781, $p < 0.001$). The heterogeneous-residual-variance Gaussian model failed to converge. Given the bounded percentage outcome, substantial concentration at 100%, and an observation at 0%, an ordered beta mixed-effects model was fitted with timepoint as a fixed effect and patient as a random intercept. The overall timepoint effect was not statistically significant at the 5% level (likelihood-ratio $\chi^2(3) = 7.08$, $p = 0.069$). None of the six pairwise comparisons was statistically significant after Holm–Šidák adjustment; the smallest adjusted p-value was 0.058 for 3M versus 1Y.

p16+EPC/EPC (%) – Lymphoma (HC)

```
> shapiro.test(resid_raw_equal)
Shapiro-Wilk normality test
data:  resid_raw_equal
W = 0.79201, p-value = 3.006e-06

> # Shapiro-Wilk test for heterogeneous-variance model
> shapiro.test(resid_raw_hetero)
```

Shapiro-Wilk normality test
data: resid_raw_hetero
W = 0.87276, p-value = 0.0002426
Conclusion: Shapiro–Wilk tests indicated departures from normality.

> print(overall_timepoint_test)
Data: dat
Models:
model_ordbeta_null: Proportion ~ (1 | Patient), zi=~0, disp=~1
model_ordbeta: Proportion ~ Timepoint + (1 | Patient), zi=~0, disp=~1

	Df	AIC	BIC	logLik	deviance	Chisq	Chi Df	Pr(>Chisq)
model_ordbeta_null	5	56.232	64.920	-23.116	46.232			
model_ordbeta	9	55.512	71.151	-18.756	37.512	8.72	4	0.06849 .

Conclusion: There is no significant difference across the timepoints.

> print(pairwise_results)

contrast	estimate	SE	df	z.ratio	p.value	p_holm_sidak	significant_0.05
Pre - End	-0.4175212	0.4784213	Inf	-0.873	0.3828	0.7703245	FALSE
Pre - 3M	-0.9135019	0.5377149	Inf	-1.699	0.0893	0.4806326	FALSE
Pre - 1Y	0.8263464	0.5872721	Inf	1.407	0.1594	0.6471933	FALSE
Pre - HC	0.5578600	0.7675537	Inf	0.727	0.4673	0.7703245	FALSE
End - 3M	-0.4959807	0.4862521	Inf	-1.020	0.3077	0.7703245	FALSE
End - 1Y	1.2438677	0.5924242	Inf	2.100	0.0358	0.2794597	FALSE
End - HC	0.9753812	0.7565062	Inf	1.289	0.1973	0.6667232	FALSE
3M - 1Y	1.7398483	0.5968457	Inf	2.915	0.0036	0.0349969	TRUE
3M - HC	1.4713619	0.8298547	Inf	1.773	0.0762	0.4696802	FALSE
1Y - HC	-0.2684865	0.8320266	Inf	-0.323	0.7469	0.7703245	FALSE

AnnexinV+EPC/EPC (%) – Lymphoma (without HC)

> shapiro.test(resid_raw_equal)
Shapiro-Wilk normality test
data: resid_raw_equal
W = 0.97267, p-value = 0.5029

> # Shapiro-Wilk test for heterogeneous-variance model
> shapiro.test(resid_raw_hetero)
Shapiro-Wilk normality test
data: resid_raw_hetero
W = 0.97386, p-value = 0.5401

Conclusion: Shapiro–Wilk tests indicated no departures from normality.

> AIC(model_raw_equal, model_raw_hetero)

	df	AIC
model_raw_equal	6	313.6899
model_raw_hetero	9	318.4070

Conclusion: Model_raw_equal is favourable.

> # Overall test: are the four timepoint means equal?
> anova(final_model, type = "marginal")

	numDF	denDF	F-value	p-value
(Intercept)	1	21	20.103590	0.0002
Timepoint	3	21	4.579999	0.0128

Conclusion: there is significant difference across the timepoints.

AnnexinV+EPC/EPC (%) – Lymphoma (HC)

> # Shapiro-Wilk test for equal-variance model
> shapiro.test(resid_raw_equal)
Shapiro-Wilk normality test
data: resid_raw_equal
W = 0.98615, p-value = 0.8835

> # Shapiro-Wilk test for heterogeneous-variance model
> shapiro.test(resid_raw_hetero)
Shapiro-Wilk normality test
data: resid_raw_hetero
W = 0.96945, p-value = 0.3164
Conclusion: Shapiro–Wilk tests indicated no departures from normality.

> AIC(model_raw_equal, model_raw_hetero)

	df	AIC
model_raw_equal	7	366.5607

```
model_raw_hetero 11 371.2782

> final_model <- model_raw_equal
> # Overall test: are the four timepoint means equal?
> anova(final_model, type = "marginal")
            numDF denDF  F-value p-value
(Intercept)     1    20 5.125956  0.0348
Timepoint       4    20 2.688127  0.0609
```

Conclusion: No, there is no significant difference across the timepoints.

```
> # All six pairwise comparisons
> summary(
+   pairs(emm, adjust = "tukey"),
+   infer = c(TRUE, TRUE)
+ )
 contrast  estimate    SE df lower.CL upper.CL t.ratio p.value
 Pre - End   -21.66 10.60 21    -53.3     10.0  -2.036  0.2841
 Pre - 3M    -27.85 11.40 21    -61.7      6.0  -2.451  0.1406
 Pre - 1Y    -35.89 11.50 21    -70.2     -1.6  -3.118  0.0374
 Pre - HC    -34.85 15.80 16    -83.1     13.4  -2.211  0.2253
 End - 3M     -6.20  8.17 21    -30.5     18.1  -0.758  0.9396
 End - 1Y    -14.23  8.48 21    -39.5     11.0  -1.679  0.4674
 End - HC    -13.19 13.90 16    -55.8     29.5  -0.947  0.8741
 3M - 1Y      -8.04  8.69 21    -33.9     17.9  -0.925  0.8841
 3M - HC      -6.99 14.20 16    -50.5     36.6  -0.492  0.9870
 1Y - HC       1.04 14.40 16    -43.1     45.1   0.073  1.0000
Degrees-of-freedom method: containment
Confidence level used: 0.95
Conf-level adjustment: tukey method for comparing a family of 5 estimates
P value adjustment: tukey method for comparing a family of 5 estimates
```

hsTnT (pg/mL) – Lymphoma

```
> shapiro.test(resid_raw_equal)
Shapiro-Wilk normality test
data:  resid_raw_equal
W = 0.83937, p-value = 0.0002004

> # Shapiro-Wilk test for heterogeneous-variance model
> shapiro.test(resid_raw_hetero)
Shapiro-Wilk normality test
data:  resid_raw_hetero
W = 0.84226, p-value = 0.0002311
```

Conclusion: Shapiro–Wilk tests indicated departures from normality in the normalized residuals.

After log-transformation,

```
> anova(model_log_equal, model_log_hetero)
                 Model df      AIC      BIC    logLik   Test  L.Ratio p-value
model_log_equal      1  6 63.53127 71.73505 -25.76564
model_log_hetero     2  9 61.26241 73.56807 -21.63120 1 vs 2 8.268865  0.0408

>shapiro.test(resid_log_equal)
Shapiro-Wilk normality test
data:  resid_log_equal
W = 0.93275, p-value = 0.04192

> shapiro.test(resid_log_hetero)
Shapiro-Wilk normality test
data:  resid_log_hetero
W = 0.94667, p-value = 0.1063
```

Conclusion: model_log_hetero is favourable.

```
>   final_model <- model_log_hetero
> # Overall timepoint effect
> anova(final_model, type = "marginal")
            numDF denDF   F-value p-value
(Intercept)     1    21 238.62808  <.0001
Timepoint       3    21  10.25173   2e-04
```

Conclusion: yes, there is significant difference across the timepoints.

```
> results$p_holm_sidak <- holm_sidak(results$p.value)
> results$significant_0.05 <- results$p_holm_sidak < 0.05
> print(results)
 contrast      ratio        SE df null t.ratio p.value p_holm_sidak significant_0.05
 Pre / End 0.4616520 0.1018224 21    1  -3.504  0.0021    0.0105060  TRUE
```

```
 Pre / 3M  0.5401971 0.0760832 21    1  -4.372  0.0003    0.0015992  TRUE
 Pre / 1Y  0.8885667 0.1838455 21    1  -0.571  0.5740    0.7973580 FALSE
 End / 3M  1.1701393 0.3024974 21    1   0.608  0.5498    0.7973580 FALSE
 End / 1Y  1.9247545 0.5767867 21    1   2.185  0.0404    0.1519210 FALSE
 3M / 1Y   1.6448934 0.4062271 21    1   2.015  0.0569    0.1610759 FALSE
```

(NTproBNP (pg/mL)) – Lymphoma

```
> shapiro.test(resid_raw_equal)
Shapiro-Wilk normality test
data:  resid_raw_equal
W = 0.5838, p-value = 1.697e-08

> # Shapiro-Wilk test for heterogeneous-variance model
> shapiro.test(resid_raw_hetero)
Shapiro-Wilk normality test
data:  resid_raw_hetero
W = 0.78667, p-value = 1.816e-05
```

Conclusion: Shapiro–Wilk tests indicated departures from normality in the normalized residuals.

```
> anova(model_log_equal, model_log_hetero)
                Model df      AIC      BIC    logLik   Test  L.Ratio p-value
model_log_equal      1  6 102.5574 110.7612 -45.27871
model_log_hetero     2  9 102.2747 114.5804 -42.13736 1 vs 2 6.282712  0.0986

> shapiro.test(resid_log_equal)
Shapiro-Wilk normality test
data:  resid_log_equal
W = 0.9833, p-value = 0.8787

> shapiro.test(resid_log_hetero)
Shapiro-Wilk normality test
data:  resid_log_hetero
W = 0.95773, p-value = 0.2228
```

Conclusion: model_log_equal is favourable.

```
>   final_model <- model_log_equal
> # Overall timepoint effect
> anova(final_model, type = "marginal")
            numDF denDF   F-value p-value
(Intercept)     1    21 183.74349  <.0001
Timepoint       3    21   3.13585  0.0471

> results$p_holm_sidak <- holm_sidak(results$p.value)
> results$significant_0.05 <- results$p_holm_sidak < 0.05
> print(results)
 contrast     ratio        SE df null t.ratio p.value p_holm_sidak significant_0.05
 Pre / End 2.020679 0.8960547 21    1   1.586  0.1276    0.4470139 FALSE
 Pre / 3M  2.087991 0.9259041 21    1   1.660  0.1117    0.4470139 FALSE
 Pre / 1Y  4.592605 2.3001288 21    1   3.044  0.0062    0.0364570  TRUE
 End / 3M  1.033312 0.4582144 21    1   0.074  0.9418    0.9417916 FALSE
 End / 1Y  2.272803 1.1382952 21    1   1.639  0.1160    0.4470139 FALSE
 3M / 1Y   2.199533 1.1015988 21    1   1.574  0.1305    0.4470139 FALSE
```
