# Supplementary material for: Compromised Endothelial Regeneration as a Potential Contributor to Long-Term Cardiovascular Complications in Childhood Cancer Survivors
Source: Cells. 2026 Nov 9;15(21):2040. doi: 10.3390/cells15212040 (PMC13761598; doi:10.3390/cells15212040)
Supplement: Supplementary file 1 [file cells-15-02040-s001.zip › Supplemental Appendix for cells.pdf]

Compromised Endothelial Regeneration as a Potential Contributor to Long-Term Cardiovascular Complications in Child-hood Cancer Survivors

Table S1: Summary of CEC and EPC counts of leukemia participants

Table S2: Summary of CEC and EPC counts of Lymphoma participants

Table S3: Intra-assay coefficient of variation (CV).

Figure S1: Longitudinal %LVEF

Figure S2: Leukemia Spearman test

Figure S3: Lymphoma Spearman test

Figure S4: Generating compensation matrix for CEC and EPC detection

Figure S5: Compensation matrix for EPC characterization

Supplemental Tables

Table S1: Summary of CEC and EPC counts of leukemia participants

	Pre-treatment	End-treatment	3M post-treatment	1Y post-treatment	Healthy
**%CEC (Relative to 10^4 PBMCs)**	1.24±0.28	0.565±0.18	0.361±0.14	0.481±0.11	0.338±0.0946
**%EPC (Relative to 10^4 PBMCs)**	1.66±0.4	1.24±0.27	0.94±0.3	0.605±0.16	1.6±0.27
**p16 %EPC (Relative to total EPCs)**	76.4±5.76	85.3±3.3	82.5±3.8	73.4±6.3	78.7±5.0
**Annexin %EPC (Relative to total EPCs)**	57.3±4.87	56.8±5.0	43.8±4.7	50.4±4.58	59.6±9.9

Table S2: Summary of CEC and EPC counts of lymphoma participants

	Pre-treatment	End-treatment	3M post-treatment	1Y post-treatment	Healthy
**%CEC (Relative to 10^4 PBMCs)**	1.74±0.6	0.249±0.1	0.702±0.28	0.755±0.3	0.32±0.085
**%EPC (Relative to 10^4 PBMCs)**	2.63±0.79	0.262±0.063	0.482±0.12	0.373±0.066	1.55±0.238
**p16 %EPC (Relative to total EPCs)**	86.7±4.9	92.1±3.4	95.7±2.1	73.5±15.3	78.7±5.0
**Annexin %EPC (Relative to total EPCs)**	22.9±7.8	46.4±9.7	53.6±9.3	62.5±8.6	59.6±9.9

Table S3: Intra-assay coefficient of variation (CV). Intra-assay CV were calculated from 9 replicates.

Cell population	Intra-assay CV, %
**%CECs (per 10^4 PBMCs)**	6.23
**%EPCs (per 10^4 PBMCs)**	22.0

Supplemental Figures

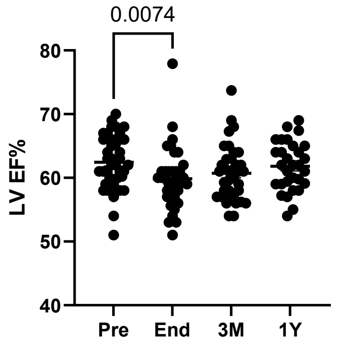

Figure S1: %LVEF of patients at Pre-treatment (n44), End-treatment (n=37), 3 months post-treatment (n=36), 1-year post-treatment (n=30). Mixed-effect analysis across the four timepoints (Pre-, End-, 3M-, 1Y) showed that the fixed effect (type III) was statistically significant (p=0.009). Šídák's multiple comparisons test demonstrated that the %LVEF is significantly lower at end-treatment when compared to pre-treatment.

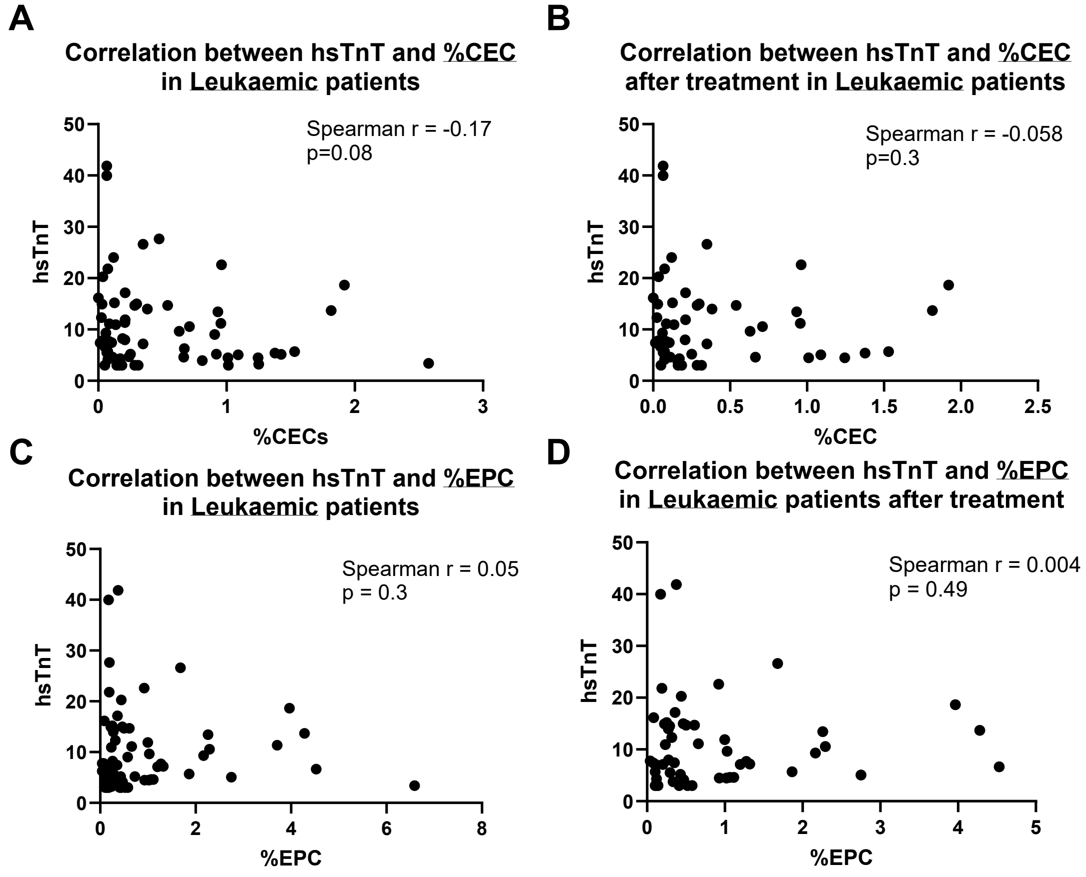

Figure S2: **Correlation between hsTnT and %CEC in Leukemic patients (A)** From Pre- to 1Y post-treatment. **(B)** After treatment (i.e., End- to 1Y post-treatment). **Correlation between hsTnT and %EPC in Leukemic patients (C)** From Pre- to 1Y post-treatment. **(D)** After treatment (i.e., End- to 1Y post-treatment). Data is presented as a scatter X-Y plot. Kolmogorov-Smirnov test determined that the populations are nonparametric. Thus, Spearman test was used to examine the correlation.

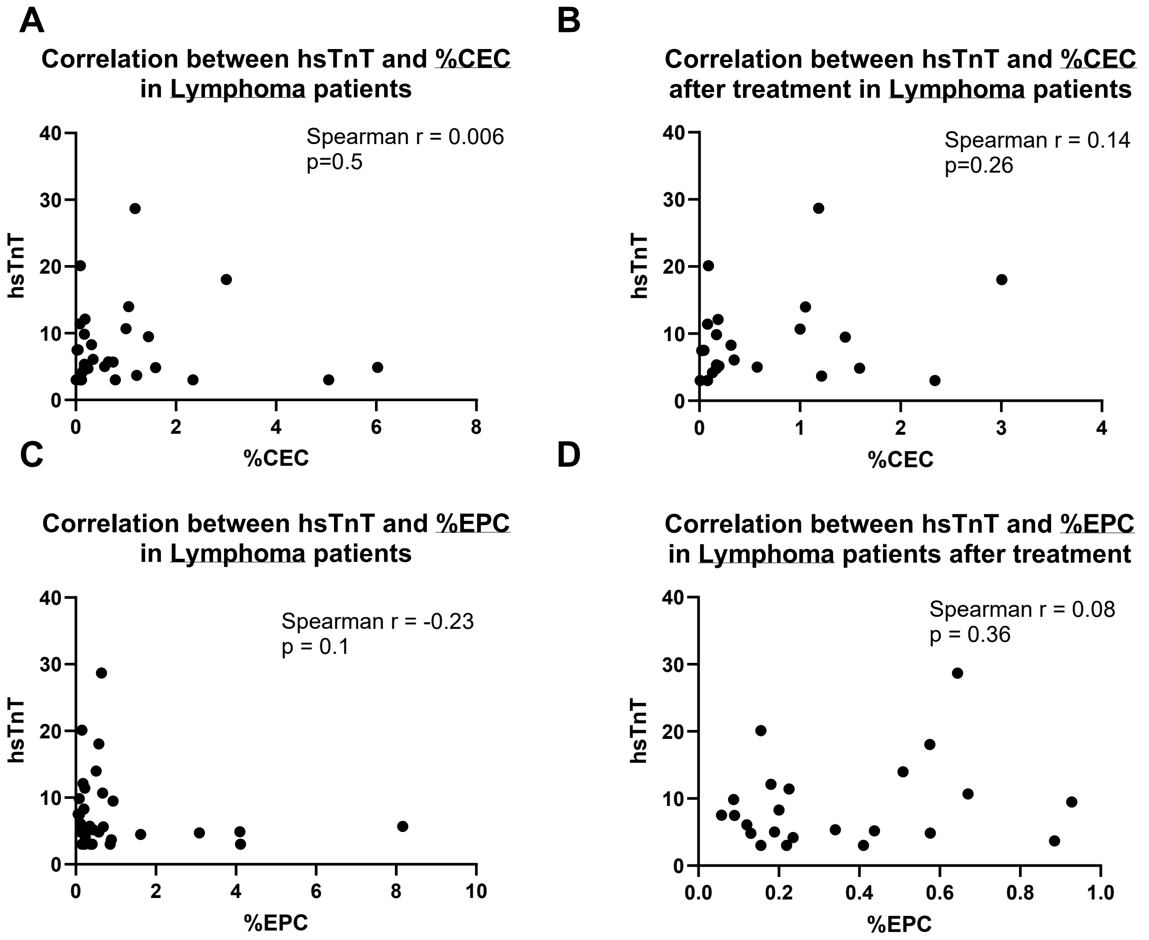

Figure S3: Correlation between hsTnT and %CEC in Lymphoma patients (A) From Pre- to 1Y post-treatment. **(B)** After treatment (i.e., End- to 1Y post-treatment). **Correlation between hsTnT and %EPC in Lymphoma patients (C)** From Pre- to 1Y post-treatment. **(D)** After treatment (i.e., End- to 1Y post-treatment). Data is presented as a scatter X-Y plot. Kolmogorov-Smirnov test determined that the populations are nonparametric. Thus, Spearman test was used to examine the correlation.

Figure S4: Generating compensation matrix for CEC and EPC detection

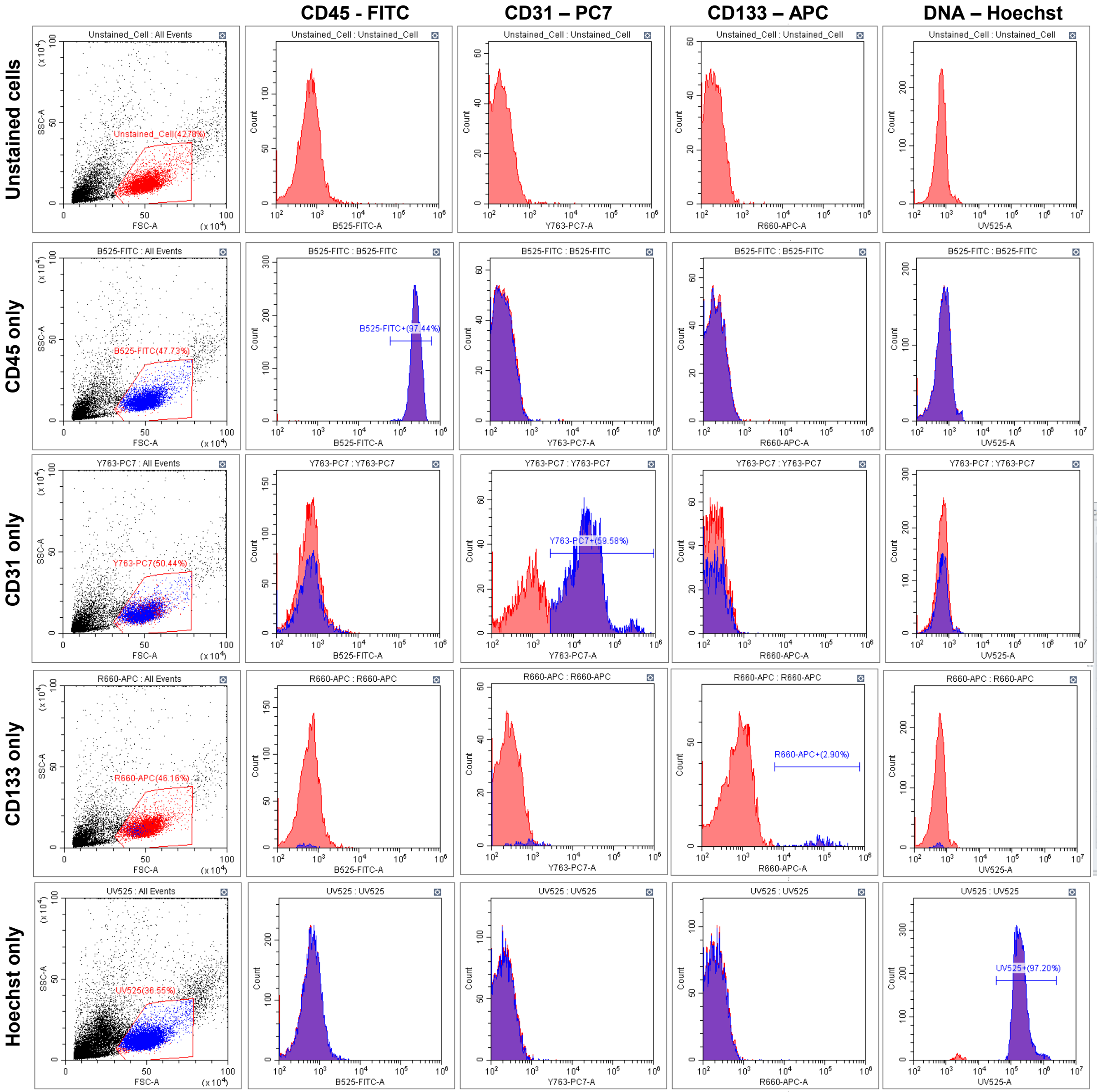

Compensation matrix:

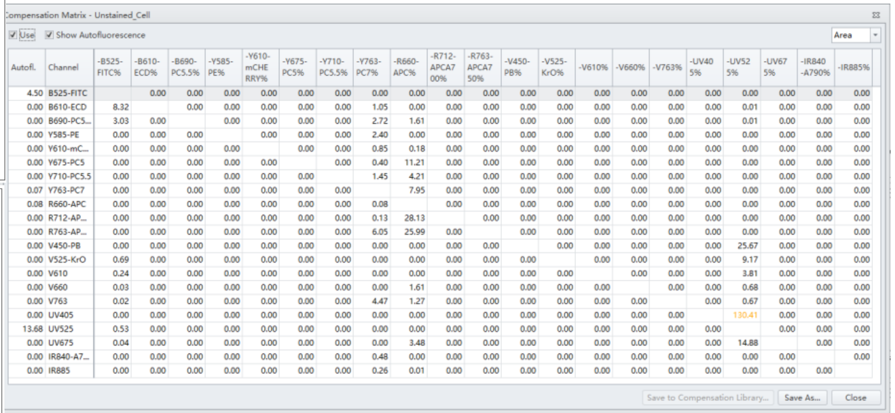

Compensation Matrix - Unstained_Cell

Use | Show Autofluorescence | Area

Autofl.	Channel	-B525-FITC%	-B610-ECD%	-B690-PC5.5%	-Y585-PE%	-Y610-mCHERRY%	-Y675-PC5%	-Y710-PC5.5%	-Y763-PC7%	-R660-APC%	-R712-APCA700%	-R763-APCA750%	-V450-PB%	-V525-KrO%	-V610%	-V660%	-V763%	-UV405%	-UV525%	-UV675%	-IR840-A790%	-IR885%
4.50	B525-FITC		0.00	0.00	0.00	0.00	0.00	0.00	0.00	0.00	0.00	0.00	0.00	0.00	0.00	0.00	0.00	0.00	0.00	0.00	0.00	0.00
0.00	B610-ECD	8.32		0.00	0.00	0.00	0.00	0.00	1.05	0.00	0.00	0.00	0.00	0.00	0.00	0.00	0.00	0.00	0.01	0.00	0.00	0.00
0.00	B690-PC5...	3.03	0.00		0.00	0.00	0.00	0.00	2.72	1.61	0.00	0.00	0.00	0.00	0.00	0.00	0.00	0.00	0.01	0.00	0.00	0.00
0.00	Y585-PE	0.00	0.00	0.00		0.00	0.00	0.00	2.40	0.00	0.00	0.00	0.00	0.00	0.00	0.00	0.00	0.00	0.00	0.00	0.00	0.00
0.00	Y610-mC...	0.00	0.00	0.00	0.00		0.00	0.00	0.85	0.18	0.00	0.00	0.00	0.00	0.00	0.00	0.00	0.00	0.00	0.00	0.00	0.00
0.00	Y675-PC5	0.00	0.00	0.00	0.00	0.00		0.00	0.40	11.21	0.00	0.00	0.00	0.00	0.00	0.00	0.00	0.00	0.00	0.00	0.00	0.00
0.00	Y710-PC5.5	0.00	0.00	0.00	0.00	0.00	0.00		1.45	4.21	0.00	0.00	0.00	0.00	0.00	0.00	0.00	0.00	0.00	0.00	0.00	0.00
0.07	Y763-PC7	0.00	0.00	0.00	0.00	0.00	0.00	0.00		7.95	0.00	0.00	0.00	0.00	0.00	0.00	0.00	0.00	0.00	0.00	0.00	0.00
0.08	R660-APC	0.00	0.00	0.00	0.00	0.00	0.00	0.00	0.08		0.00	0.00	0.00	0.00	0.00	0.00	0.00	0.00	0.00	0.00	0.00	0.00
0.00	R712-AP...	0.00	0.00	0.00	0.00	0.00	0.00	0.00	0.13	28.13		0.00	0.00	0.00	0.00	0.00	0.00	0.00	0.00	0.00	0.00	0.00
0.00	R763-AP...	0.00	0.00	0.00	0.00	0.00	0.00	0.00	6.05	25.99	0.00		0.00	0.00	0.00	0.00	0.00	0.00	0.00	0.00	0.00	0.00
0.00	V450-PB	0.00	0.00	0.00	0.00	0.00	0.00	0.00	0.00	0.00	0.00	0.00		0.00	0.00	0.00	0.00	0.00	25.67	0.00	0.00	0.00
0.00	V525-KrO	0.69	0.00	0.00	0.00	0.00	0.00	0.00	0.00	0.00	0.00	0.00	0.00		0.00	0.00	0.00	0.00	9.17	0.00	0.00	0.00
0.00	V610	0.24	0.00	0.00	0.00	0.00	0.00	0.00	0.00	0.00	0.00	0.00	0.00	0.00		0.00	0.00	0.00	3.81	0.00	0.00	0.00
0.00	V660	0.03	0.00	0.00	0.00	0.00	0.00	0.00	0.00	1.61	0.00	0.00	0.00	0.00	0.00		0.00	0.00	0.68	0.00	0.00	0.00
0.00	V763	0.02	0.00	0.00	0.00	0.00	0.00	0.00	4.47	1.27	0.00	0.00	0.00	0.00	0.00	0.00		0.00	0.67	0.00	0.00	0.00
0.00	UV405	0.00	0.00	0.00	0.00	0.00	0.00	0.00	0.00	0.00	0.00	0.00	0.00	0.00	0.00	0.00	0.00		130.41	0.00	0.00	0.00
13.68	UV525	0.53	0.00	0.00	0.00	0.00	0.00	0.00	0.00	0.00	0.00	0.00	0.00	0.00	0.00	0.00	0.00	0.00		0.00	0.00	0.00
0.00	UV675	0.04	0.00	0.00	0.00	0.00	0.00	0.00	0.00	3.48	0.00	0.00	0.00	0.00	0.00	0.00	0.00	0.00	14.88		0.00	0.00
0.00	IR840-A7...	0.00	0.00	0.00	0.00	0.00	0.00	0.00	0.48	0.00	0.00	0.00	0.00	0.00	0.00	0.00	0.00	0.00	0.00	0.00		0.00
0.00	IR885	0.00	0.00	0.00	0.00	0.00	0.00	0.00	0.26	0.01	0.00	0.00	0.00	0.00	0.00	0.00	0.00	0.00	0.00	0.00	0.00	

Save to Compensation Library... | Save As... | Close

Figure S5: Generating compensation matrix for EPC characterisation

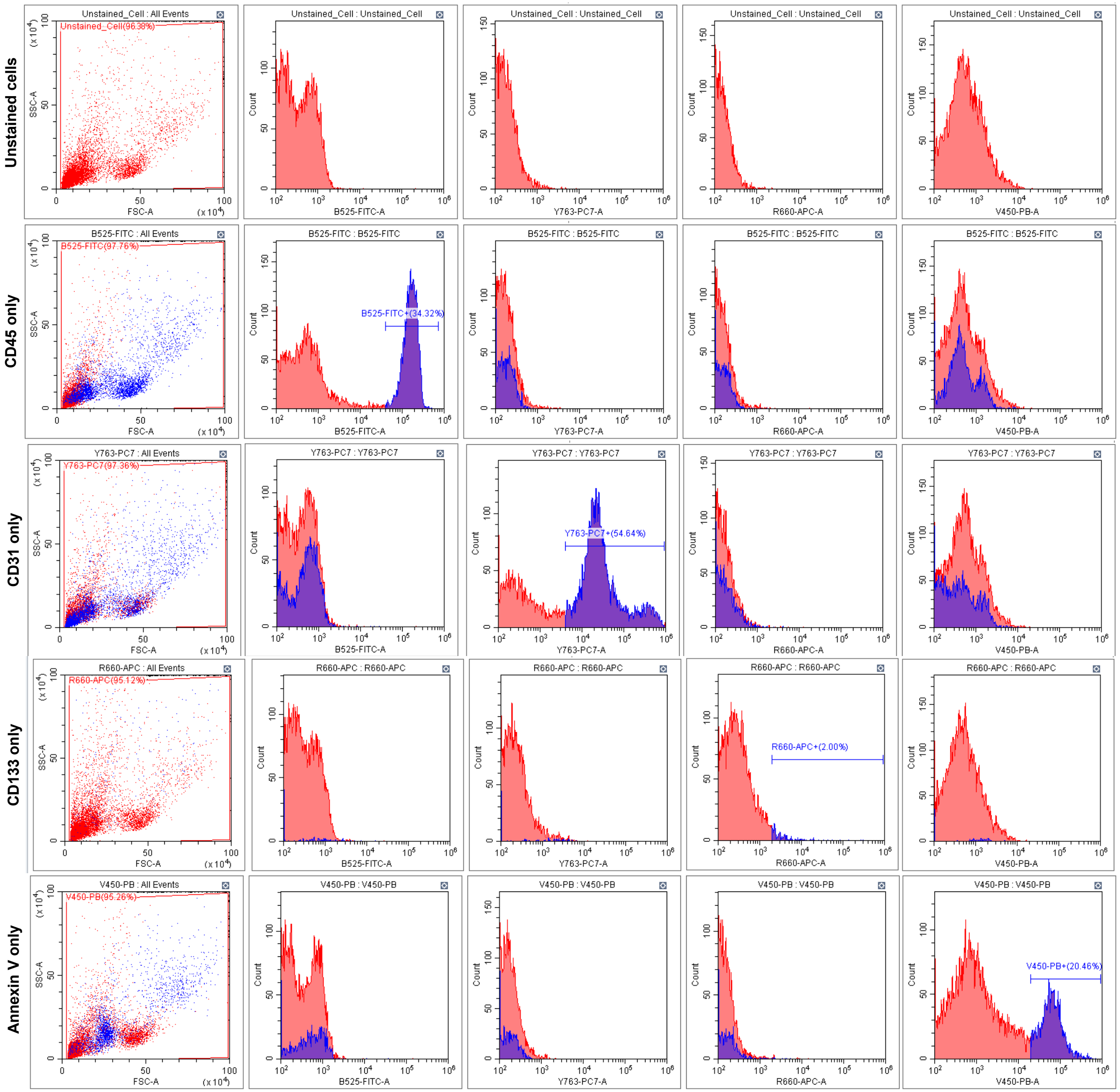

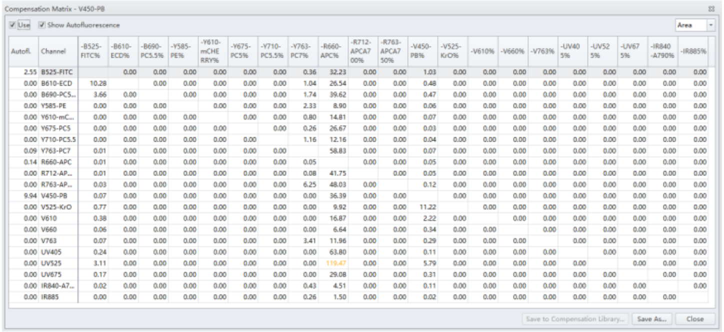

Compensation Matrix - V450-PB

Use | Show Autofluorescence | Area

Autofl.	Channel	-B525-FITC%	-B610-ECD%	-B690-PC5.5%	-Y585-PE%	-Y610-mCHERRY%	-Y675-PC5%	-Y710-PC5.5%	-Y763-PC7%	-R660-APC%	-R712-APCA700%	-R763-APCA750%	-V450-PB%	-V525-KrO%	-V610%	-V660%	-V763%	-UV405%	-UV525%	-UV675%	-IR840-A790%	-IR885%
2.55	B525-FITC		0.00	0.00	0.00	0.00	0.00	0.00	0.36	32.23	0.00	0.00	1.03	0.00	0.00	0.00	0.00	0.00	0.00	0.00	0.00	0.00
0.00	B610-ECD	10.28		0.00	0.00	0.00	0.00	0.00	1.04	26.54	0.00	0.00	0.48	0.00	0.00	0.00	0.00	0.00	0.00	0.00	0.00	0.00
0.00	B690-PC5...	3.66	0.00		0.00	0.00	0.00	0.00	1.74	39.62	0.00	0.00	0.47	0.00	0.00	0.00	0.00	0.00	0.00	0.00	0.00	0.00
0.00	Y585-PE	0.00	0.00	0.00		0.00	0.00	0.00	2.33	8.90	0.00	0.00	0.06	0.00	0.00	0.00	0.00	0.00	0.00	0.00	0.00	0.00
0.00	Y610-mC...	0.00	0.00	0.00	0.00		0.00	0.00	0.80	14.81	0.00	0.00	0.07	0.00	0.00	0.00	0.00	0.00	0.00	0.00	0.00	0.00
0.00	Y675-PC5	0.00	0.00	0.00	0.00	0.00		0.00	0.26	26.67	0.00	0.00	0.03	0.00	0.00	0.00	0.00	0.00	0.00	0.00	0.00	0.00
0.00	Y710-PC5.5	0.00	0.00	0.00	0.00	0.00	0.00		1.16	12.16	0.00	0.00	0.04	0.00	0.00	0.00	0.00	0.00	0.00	0.00	0.00	0.00
0.09	Y763-PC7	0.01	0.00	0.00	0.00	0.00	0.00	0.00		58.83	0.00	0.00	0.07	0.00	0.00	0.00	0.00	0.00	0.00	0.00	0.00	0.00
0.14	R660-APC	0.01	0.00	0.00	0.00	0.00	0.00	0.00	0.05		0.00	0.00	0.05	0.00	0.00	0.00	0.00	0.00	0.00	0.00	0.00	0.00
0.00	R712-AP...	0.01	0.00	0.00	0.00	0.00	0.00	0.00	0.08	41.75		0.00	0.05	0.00	0.00	0.00	0.00	0.00	0.00	0.00	0.00	0.00
0.00	R763-AP...	0.03	0.00	0.00	0.00	0.00	0.00	0.00	6.25	48.03	0.00		0.12	0.00	0.00	0.00	0.00	0.00	0.00	0.00	0.00	0.00
9.94	V450-PB	0.07	0.00	0.00	0.00	0.00	0.00	0.00	0.00	36.39	0.00	0.00		0.00	0.00	0.00	0.00	0.00	0.00	0.00	0.00	0.00
0.00	V525-KrO	0.77	0.00	0.00	0.00	0.00	0.00	0.00	0.00	9.92	0.00	0.00	11.22		0.00	0.00	0.00	0.00	0.00	0.00	0.00	0.00
0.00	V610	0.38	0.00	0.00	0.00	0.00	0.00	0.00	0.00	16.87	0.00	0.00	2.22	0.00		0.00	0.00	0.00	0.00	0.00	0.00	0.00
0.00	V660	0.06	0.00	0.00	0.00	0.00	0.00	0.00	0.00	6.64	0.00	0.00	0.34	0.00	0.00		0.00	0.00	0.00	0.00	0.00	0.00
0.00	V763	0.07	0.00	0.00	0.00	0.00	0.00	0.00	3.41	11.96	0.00	0.00	0.29	0.00	0.00	0.00		0.00	0.00	0.00	0.00	0.00
0.00	UV405	0.24	0.00	0.00	0.00	0.00	0.00	0.00	0.00	63.80	0.00	0.00	0.11	0.00	0.00	0.00	0.00		0.00	0.00	0.00	0.00
0.00	UV525	3.11	0.00	0.00	0.00	0.00	0.00	0.00	0.00	119.47	0.00	0.00	5.79	0.00	0.00	0.00	0.00	0.00		0.00	0.00	0.00
0.00	UV675	0.17	0.00	0.00	0.00	0.00	0.00	0.00	0.00	29.08	0.00	0.00	0.31	0.00	0.00	0.00	0.00	0.00	0.00		0.00	0.00
0.00	IR840-A7...	0.02	0.00	0.00	0.00	0.00	0.00	0.00	0.43	4.51	0.00	0.00	0.11	0.00	0.00	0.00	0.00	0.00	0.00	0.00		0.00
0.00	IR885	0.00	0.00	0.00	0.00	0.00	0.00	0.00	0.26	1.50	0.00	0.00	0.02	0.00	0.00	0.00	0.00	0.00	0.00	0.00	0.00	

Save to Compensation Library... | Save As... | Close

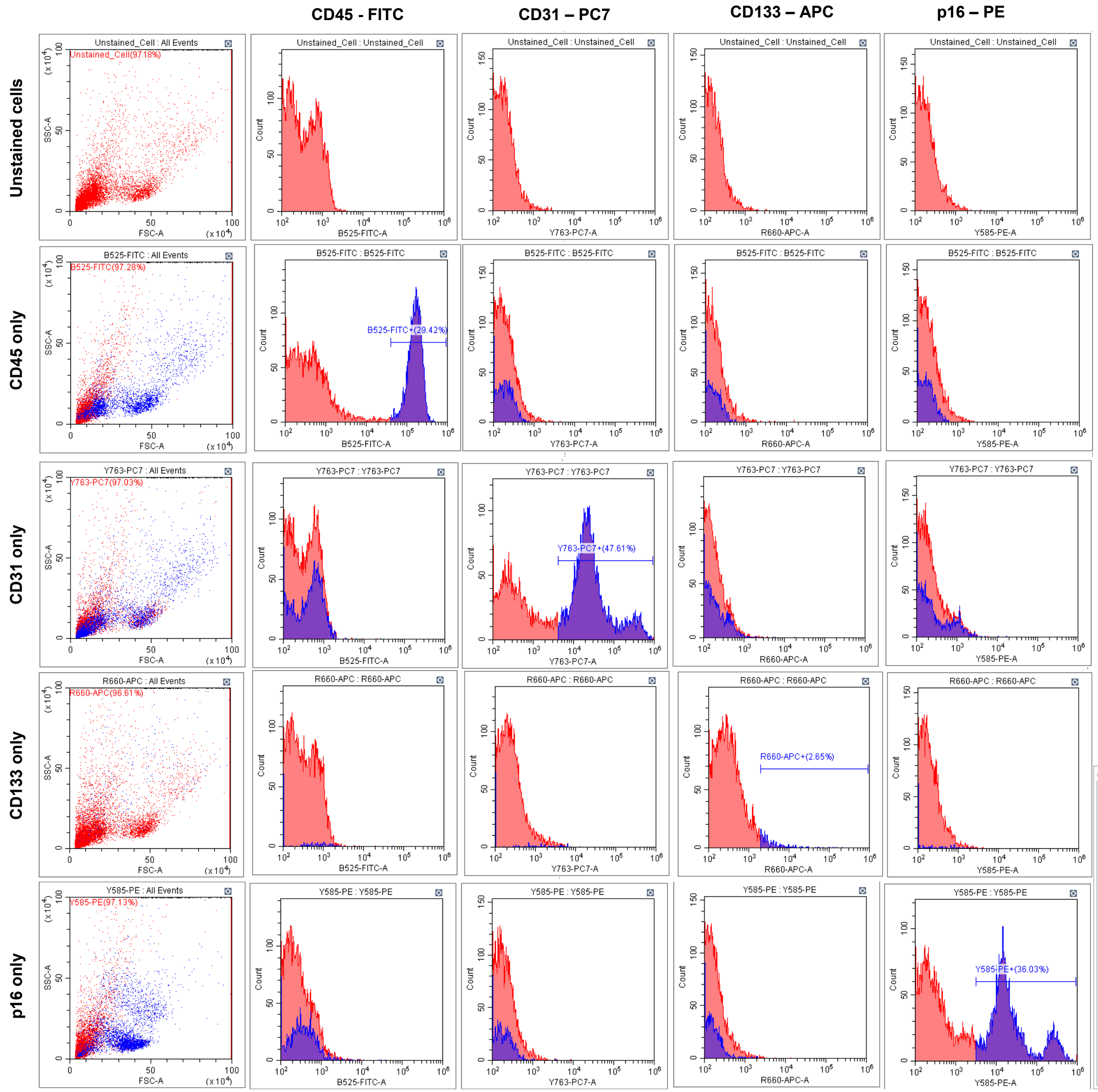

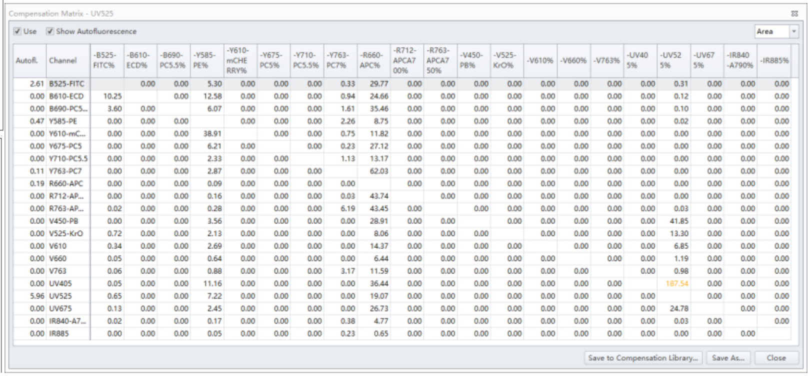
Compensation Matrix - UV525

☑ Use ☑ Show Autofluorescence — Area

Autofl.	Channel	-B525-FITC%	-B610-ECD%	-B690-PC5.5%	-Y585-PE%	-Y610-mCHERRY%	-Y675-PC5%	-Y710-PC5.5%	-Y763-PC7%	-R660-APC%
2.61	B525-FITC		0.00	0.00	5.30	0.00	0.00	0.00	0.33	29.77
0.00	B610-ECD	10.25		0.00	12.58	0.00	0.00	0.00	0.94	24.66
0.00	B690-PC5...	3.60	0.00		6.07	0.00	0.00	0.00	1.61	35.46
0.47	Y585-PE	0.00	0.00	0.00		0.00	0.00	0.00	2.26	8.75
0.00	Y610-mC...	0.00	0.00	0.00	38.91		0.00	0.00	0.75	11.82
0.00	Y675-PC5	0.00	0.00	0.00	6.21	0.00		0.00	0.23	27.12
0.00	Y710-PC5.5	0.00	0.00	0.00	2.33	0.00	0.00		1.13	13.17
0.11	Y763-PC7	0.00	0.00	0.00	2.87	0.00	0.00	0.00		62.03
0.19	R660-APC	0.00	0.00	0.00	0.09	0.00	0.00	0.00	0.00	
0.00	R712-AP...	0.00	0.00	0.00	0.16	0.00	0.00	0.00	0.03	43.74
0.00	R763-AP...	0.02	0.00	0.00	0.28	0.00	0.00	0.00	6.19	43.45
0.00	V450-PB	0.00	0.00	0.00	3.56	0.00	0.00	0.00	0.00	28.91
0.00	V525-KrO	0.72	0.00	0.00	2.13	0.00	0.00	0.00	0.00	8.06
0.00	V610	0.34	0.00	0.00	2.69	0.00	0.00	0.00	0.00	14.37
0.00	V660	0.05	0.00	0.00	0.64	0.00	0.00	0.00	0.00	6.44
0.00	V763	0.06	0.00	0.00	0.88	0.00	0.00	0.00	3.17	11.59
0.00	UV405	0.05	0.00	0.00	11.16	0.00	0.00	0.00	0.00	36.44
5.96	UV525	0.65	0.00	0.00	7.22	0.00	0.00	0.00	0.00	19.07
0.00	UV675	0.13	0.00	0.00	2.45	0.00	0.00	0.00	0.00	26.73
0.00	IR840-A7...	0.02	0.00	0.00	0.17	0.00	0.00	0.00	0.38	4.77
0.00	IR885	0.00	0.00	0.00	0.05	0.00	0.00	0.00	0.23	0.65

Channel	-R712-APCA700%	-R763-APCA750%	-V450-PB%	-V525-KrO%	-V610%	-V660%	-V763%	-UV405%	-UV525%	-UV675%	-IR840-A790%	-IR885%
B525-FITC	0.00	0.00	0.00	0.00	0.00	0.00	0.00	0.00	0.31	0.00	0.00	0.00
B610-ECD	0.00	0.00	0.00	0.00	0.00	0.00	0.00	0.00	0.12	0.00	0.00	0.00
B690-PC5...	0.00	0.00	0.00	0.00	0.00	0.00	0.00	0.00	0.10	0.00	0.00	0.00
Y585-PE	0.00	0.00	0.00	0.00	0.00	0.00	0.00	0.00	0.02	0.00	0.00	0.00
Y610-mC...	0.00	0.00	0.00	0.00	0.00	0.00	0.00	0.00	0.00	0.00	0.00	0.00
Y675-PC5	0.00	0.00	0.00	0.00	0.00	0.00	0.00	0.00	0.00	0.00	0.00	0.00
Y710-PC5.5	0.00	0.00	0.00	0.00	0.00	0.00	0.00	0.00	0.00	0.00	0.00	0.00
Y763-PC7	0.00	0.00	0.00	0.00	0.00	0.00	0.00	0.00	0.00	0.00	0.00	0.00
R660-APC	0.00	0.00	0.00	0.00	0.00	0.00	0.00	0.00	0.00	0.00	0.00	0.00
R712-AP...		0.00	0.00	0.00	0.00	0.00	0.00	0.00	0.00	0.00	0.00	0.00
R763-AP...	0.00		0.00	0.00	0.00	0.00	0.00	0.00	0.03	0.00	0.00	0.00
V450-PB	0.00	0.00		0.00	0.00	0.00	0.00	0.00	41.85	0.00	0.00	0.00
V525-KrO	0.00	0.00	0.00		0.00	0.00	0.00	0.00	13.30	0.00	0.00	0.00
V610	0.00	0.00	0.00	0.00		0.00	0.00	0.00	6.85	0.00	0.00	0.00
V660	0.00	0.00	0.00	0.00	0.00		0.00	0.00	1.19	0.00	0.00	0.00
V763	0.00	0.00	0.00	0.00	0.00	0.00		0.00	0.98	0.00	0.00	0.00
UV405	0.00	0.00	0.00	0.00	0.00	0.00	0.00		187.54	0.00	0.00	0.00
UV525	0.00	0.00	0.00	0.00	0.00	0.00	0.00	0.00		0.00	0.00	0.00
UV675	0.00	0.00	0.00	0.00	0.00	0.00	0.00	0.00	24.78		0.00	0.00
IR840-A7...	0.00	0.00	0.00	0.00	0.00	0.00	0.00	0.00	0.03	0.00		0.00
IR885	0.00	0.00	0.00	0.00	0.00	0.00	0.00	0.00	0.00	0.00	0.00	

Save to Compensation Library... Save As... Close
